# Comprehensive Study of Lanthanum Aluminate High-Dielectric-Constant Gate Oxides for AdvancedCMOS Devices

**DOI:** 10.3390/ma5030443

**Published:** 2012-03-14

**Authors:** Masamichi Suzuki

**Affiliations:** Advanced LSI Technology Laboratory, Corporate Research & Development Center, Toshiba Corporation, 8 Shinsugita-cho, Isogo-ku, Yokohama 235-8522, Japan; E-Mail: masamichi2.suzuki@toshiba.co.jp

**Keywords:** LaAlO_3_, high-*k*, gate dielectrics, metal gate, MOSFET, schottky source/drain, EOT, direct contact

## Abstract

A comprehensive study of the electrical and physical characteristics of Lanthanum Aluminate (LaAlO_3_) high-dielectric-constant gate oxides for advanced CMOS devices was performed. The most distinctive feature of LaAlO_3_ as compared with Hf-based high-*k* materials is the thermal stability at the interface with Si, which suppresses the formation of a low-permittivity Si oxide interfacial layer. Careful selection of the film deposition conditions has enabled successful deposition of an LaAlO_3_ gate dielectric film with an equivalent oxide thickness (EOT) of 0.31 nm. Direct contact with Si has been revealed to cause significant tensile strain to the Si in the interface region. The high stability of the effective work function with respect to the annealing conditions has been demonstrated through comparison with Hf-based dielectrics. It has also been shown that the effective work function can be tuned over a wide range by controlling the La/(La + Al) atomic ratio. In addition, gate-first n-MOSFETs with ultrathin EOT that use sulfur-implanted Schottky source/drain technology have been fabricated using a low-temperature process.

## 1. Introduction

High-*k* gate dielectrics have been widely researched over the last decade and are currently being used in practical devices [1]. The first generation of high-*k* materials were Hf-based dielectrics because Hf atoms have the same valence state as Si atoms (+4) and can therefore easily replace them. However, almost all Hf-based high-*k* dielectrics on Si are accompanied by an interfacial layer with low permittivity at the interface with Si.

According to the latest International Technology Roadmap for Semiconductors (ITRS) [2], an equivalent oxide thickness (EOT) of 0.5 nm will be required for high-performance logic applications in 2016 or beyond. One of the keys to achieving such a thin EOT value is suppression of interfacial layer formation, because 0.5 nm is equivalent to only a few monolayers of SiO_2_. La_2_O_3_ is known to be superior to Hf-based high-*k* materials in terms of both its thermodynamic stability on Si and its high dielectric constant (~27) [3]. However, La_2_O_3_ is so moisture sensitive that it is considered to be unsuitable for large-scale integration (LSI) processes. On the other hand, LaAlO_3_, which is a compound of La_2_O_3_ and Al_2_O_3_, has high immunity against moisture in the environment. Its thermal stability on Si is similar to that of La_2_O_3_ on Si, and its dielectric constant (25–27) [4] is nearly the same as that of La_2_O_3_. We have therefore focused on LaAlO_3_ as a candidate high-*k* material for achieving an EOT of 0.5 nm.

In this paper, we comprehensively review the electrical and physical characteristics of LaAlO_3_ gate dielectrics and demonstrate their high potential as successors to Hf-based high-*k* materials.

## 2. Ultrathin EOT and Ultralow Leakage Current Achieved through the High-Temperature Deposition Technique

In this section, LaAlO_3_ gate dielectrics directly deposited on Si substrates will be shown to have an ultrathin EOT and an ultralow leakage current, and the importance of the deposition temperature will be discussed [5].

The LaAlO_3_ films used were deposited on n-type Si (100) substrates by a pulsed laser deposition (PLD) method using a KrF excimer laser (λ = 248 nm). The deposition of LaAlO_3_ films was performed under the base pressure of the PLD chamber (4 × 10^−7^ Torr) at room temperature (RT) and at 700 °C. No gas was introduced into the chamber during deposition. The electrical characteristics were investigated using metal insulator semiconductor (MIS) capacitors, in which the Mo deposited by e-beam evaporation was used as the gate electrode material. The energy band profile of the LaAlO_3_/Si system was investigated using X-ray photoelectron spectroscopy (XPS). In order to investigate the behavior of interfacial layer formation due to oxidizing agents in the LaAlO_3_ film, annealing in a vacuum ambient was performed in the temperature range of 400 °C to 600 °C. After annealing, thermal desorption spectroscopy (TDS) analysis was performed to determine the components desorbed from the LaAlO_3_ film during the annealing process.

### 2.1. Fabrication and Electrical Characteristics of LaAlO_3_ Gate Dielectrics with Ultrathin EOT and Ultralow Leakage Current

**Figure 1:**
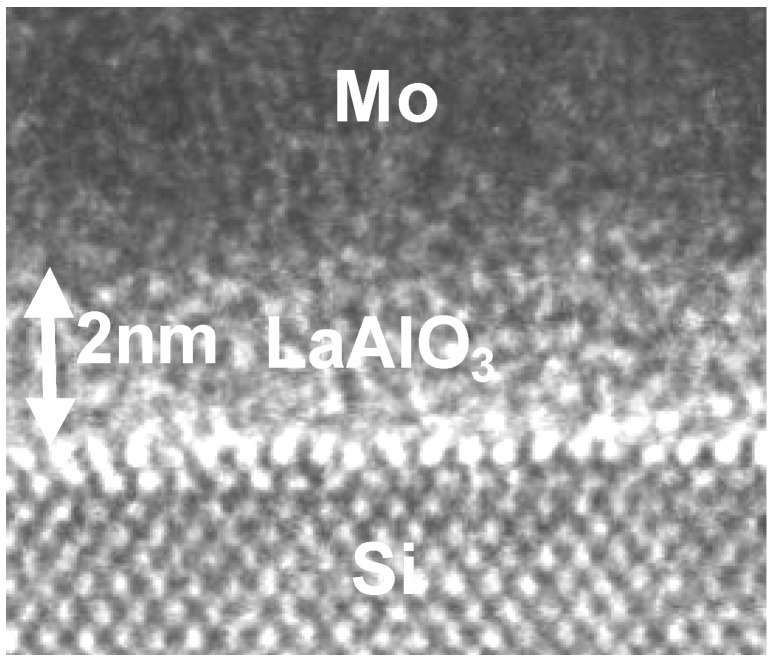
Cross-sectional TEM image of the Mo/LaAlO_3_/Si substrate gate stack.

**Figure 2:**
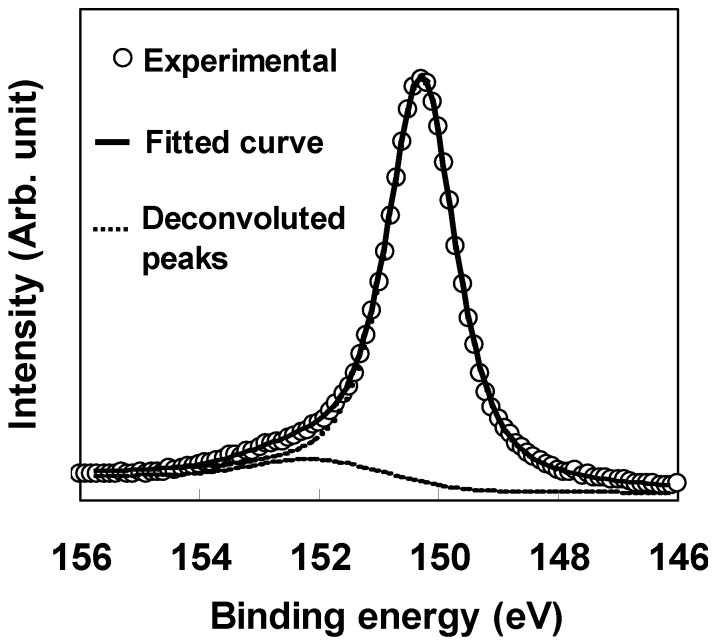
Si 2s XPS spectrum of the LaAlO_3_/Si structure and the curve fitting results.

Figure 1 shows a transmission electron microscopy (TEM) image of an Mo/LaAlO_3_/Si gate stack. The LaAlO_3_ film was deposited at 700 °C. This image shows that Si-oxide does not grow at the interface of the LaAlO_3_ film and Si substrate. In other words, the LaAlO_3_ film has been deposited directly on the Si substrate. The direct LaAlO_3_/Si interface was also confirmed by the results of XPS analysis. Figure 2 shows the Si 2s XPS spectrum of the film shown in Figure 1, obtained before the deposition of the Mo gate. The curve fitting result is also shown. The spectrum was composed of a main peak, corresponding to the Si substrate, at around 150 eV and a smaller peak, corresponding to an Si suboxide, at around 152 eV. Comparison of the areas of the deconvoluted peaks and subtraction of the background by the Shirley method [6] indicated that the small peak corresponded to SiO_2_ with a thickness of 0.2 nm, which was roughly equivalent to one monolayer of Si-O-La (Al) bonds at the interface between the LaAlO_3_ film and the Si substrate.

**Figure 3:**
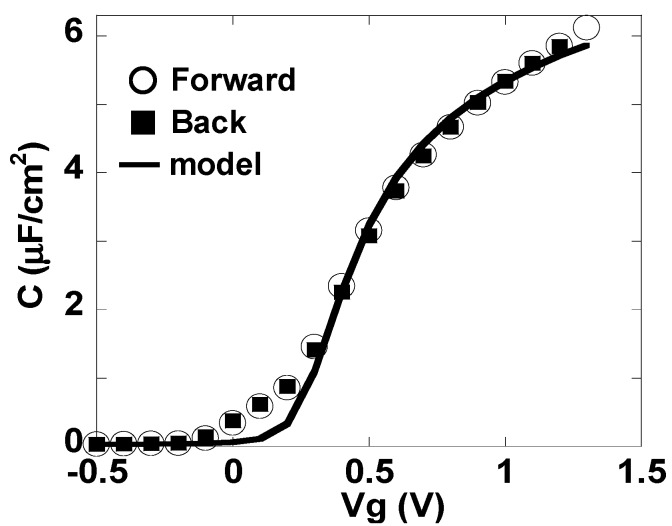
C-Vg characteristics of the metal insulator semiconductor (MIS) capacitor shown in Figure 1.

**Figure 4:**
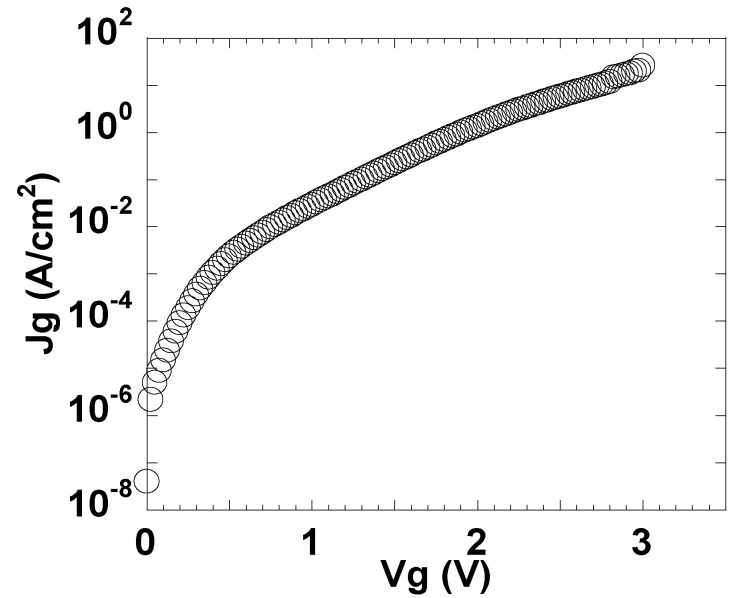
Jg-Vg characteristics of the MIS capacitor shown in Figure 1.

Figure 3 and Figure 4 respectively show the C-Vg and Jg-Vg characteristics of the MIS capacitor shown in Figure 1. The C-V curve in Figure 3 was corrected using the two-frequency method [7]. This curve exhibits negligible hysteresis and indicates that the capacitance in the accumulation condition is quite large (6 μF/cm^2^). The EOT value, estimated by comparison with the ideal C-V curve [8] shown by the solid line in Figure 3, was as small as 0.31 nm. The dielectric constant estimated from the relationship between the film thickness and the EOT was 25, which is the same as that reported for LaAlO_3_ film [4].

**Figure 5:**
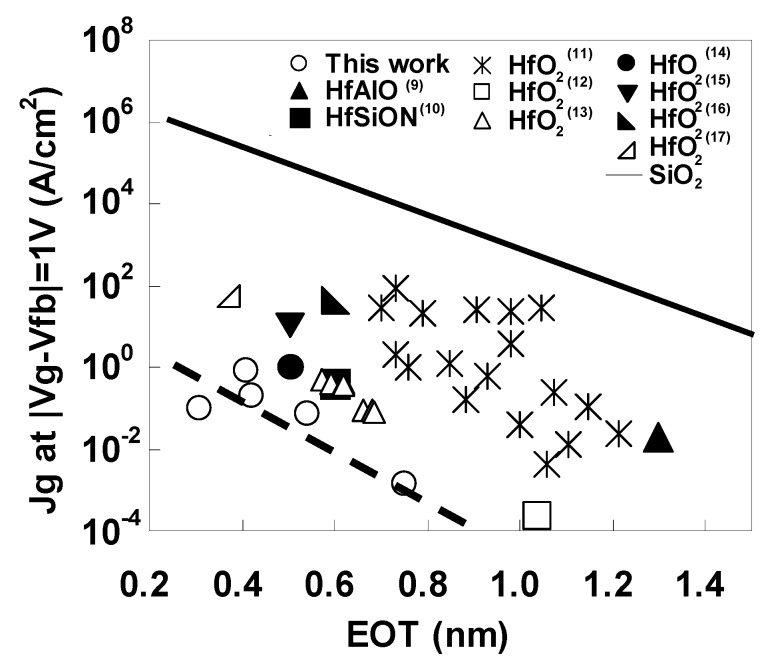
Relationship between equivalent oxide thickness (EOT) and Jg for LaAlO_3_ (this work), for Hf-based dielectrics (literature), and for SiO_2_.

The leakage current characteristics were also excellent. The gate leakage current density (Jg) at Vg = Vfb + 1 V was as low as 0.1 A/cm^2^ at EOT = 0.31 nm. Figure 5 shows plots of the relationship between Jg at |Vg − Vfb| = 1 V and EOT for LaAlO_3_ (this work) and various Hf-based dielectrics [9,10,11,12,13,14,15,16,17], and also shows the simulation results for direct tunneling current in SiO_2_. At the same EOT, the leakage current for LaAlO_3_ was six orders of magnitude lower than that for SiO_2_ and at least one order of magnitude lower than that for Hf-based dielectrics.

**Figure 6:**
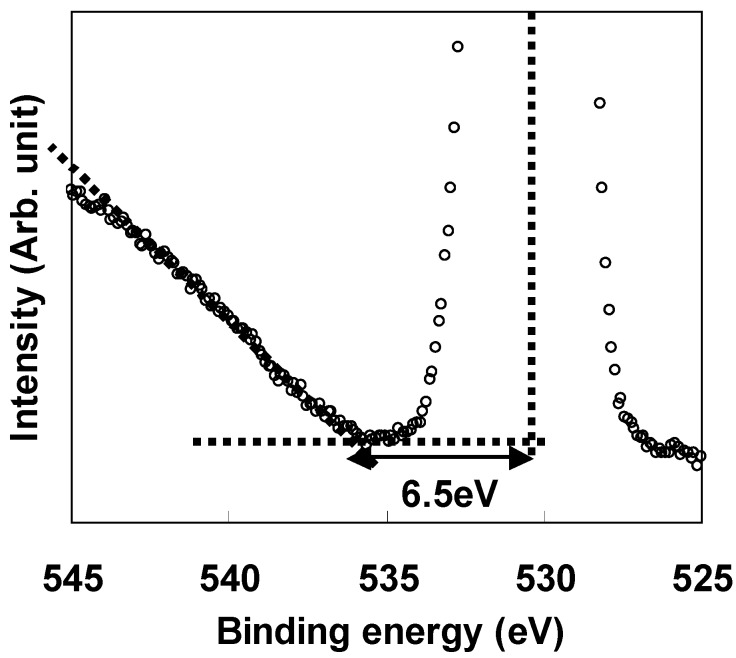
XPS O 1s loss spectrum for 10-nm LaAlO_3_ film.

The low leakage current characteristics of LaAlO_3_ in Figure 5 are thought to be partly because of the large conduction band offset (ΔEc) at the LaAlO_3_/Si interface. Therefore, the energy band profile at the LaAlO_3_/Si interface was investigated using XPS. All the measurements were performed with a photoelectron take-off angle of 90° with respect to the specimen surface. LaAlO_3_ films with thicknesses of 10 nm and 3 nm and Si substrates treated with dilute HF were prepared for the band alignment measurement. The 10-nm-thick LaAlO_3_/Si specimen was used to obtain the valence band spectra (Al 2p and O 1s) determined solely by LaAlO_3_ film, while the 3-nm-thick LaAlO_3_/Si specimen was used to evaluate the energy difference between Si 2p and Al 2p. The top of the Si 2p valence band was determined using HF-treated Si substrate. The LaAlO_3_ bandgap was evaluated using the O 1s loss spectrum in the 10-nm-thick LaAlO_3_/Si specimen. Figure 6 shows the O 1s loss spectrum for the LaAlO_3_ film. The bandgap value, which corresponds to the energy difference between the peak top energy of the O 1s spectrum and the cut-off energy of the O 1s loss spectrum, was estimated to be 6.5 eV. This value is comparable to the reported data for LaAlO_3_ films [4,18,19].

**Figure 7:**
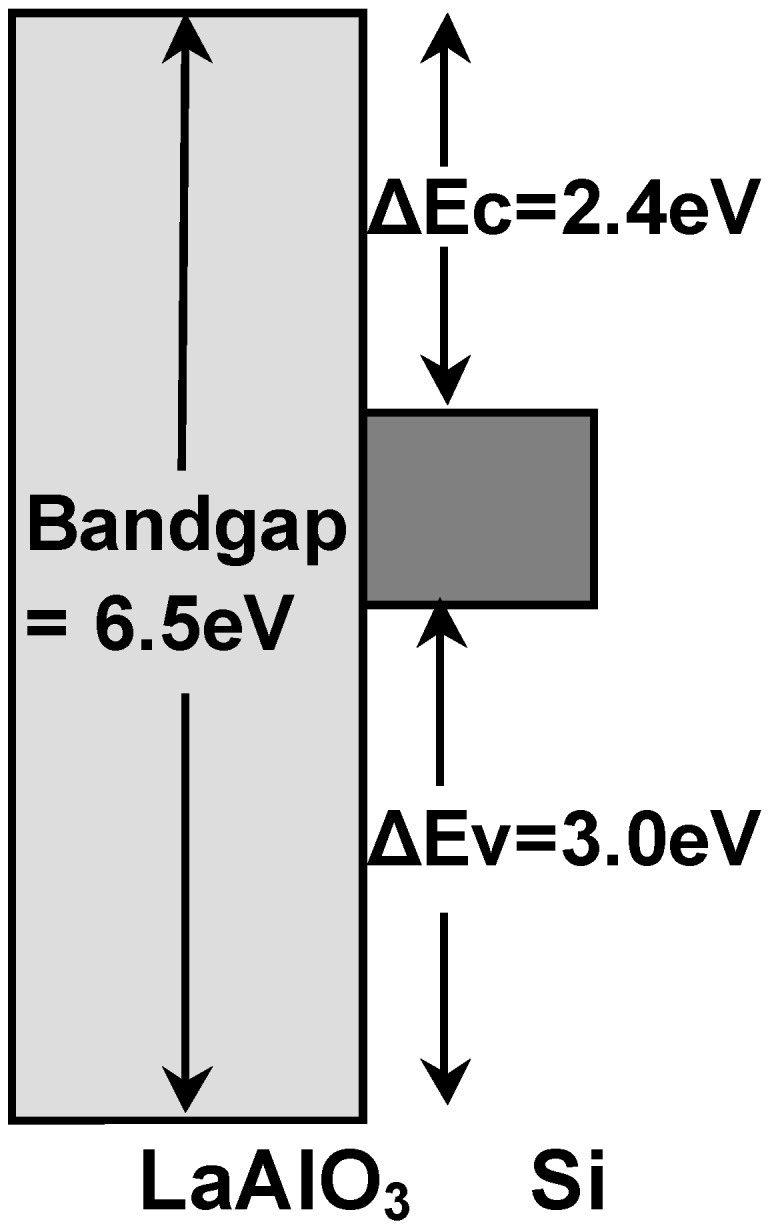
Energy-band profile of the LaAlO_3_/Si structure determined from XPS.

The valence band offset (ΔEv) was evaluated from Equation (1) below using the energies of the core spectra for Al 2p of the LaAlO_3_ film (E_Al2p_) and Si 2p of the Si substrate (E_Si2p_) [18]:

**ΔEv = (E_Si2p_ − Ev(Si))_Si-substrate_ − (E_Al2p_ − Ev(LaAlO_3_))_10-nm LaAlO3_ − (E_Si2p_ − E_Al2p_)_3-nm LaAlO3_** (1)

where Ev(Si) and Ev(LaAlO_3_) are the valence band maxima of Si and LaAlO_3_, respectively. Using this equation, ΔEv was estimated to be 3.0 eV. ΔEc was then determined to be 2.4 eV by subtracting the valence band offset (3.0 eV) and the bandgap of Si (1.1 eV) from the bandgap of LaAlO_3_ (6.5 eV). Based on the above results, the energy band profile of the LaAlO_3_/Si structure was determined to be as shown in Figure 7. The LaAlO_3_/Si direct contact interface has larger band offsets than those of the HfO_2_/Si interface (ΔEc = 1.91 eV and ΔEv = 2.22 eV [20]), leading to a lower leakage current in the LaAlO_3_/Si system than in the HfO_2_/Si system.

### 2.2. Interfacial Layer Formation during Post-Deposition Annealing due to Oxidizing Agents in LaAlO_3_ Film

**Figure 8:**
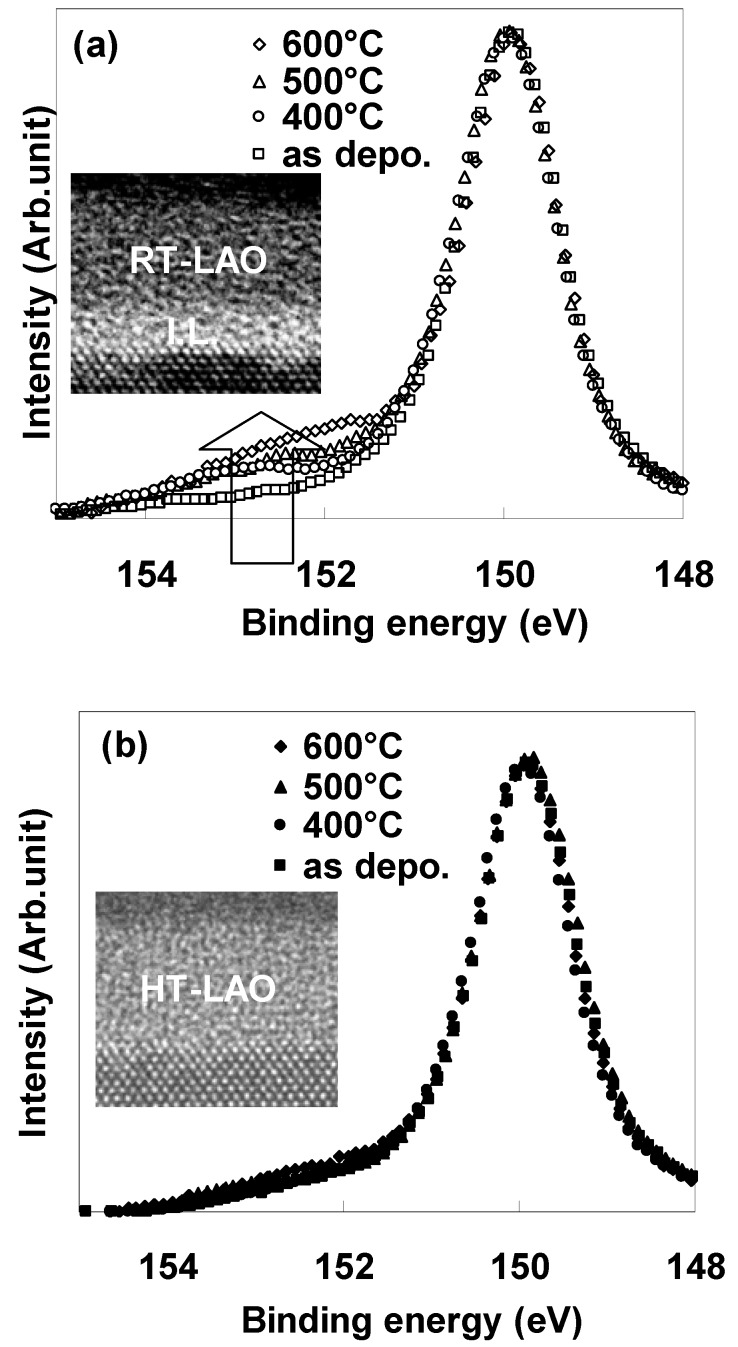
Si 2s XPS spectra for vacuum annealing temperatures ranging from 400 °C to 600 °C for (**a**) RT-LAO and (**b**) HT-LAO. The insets show TEM images after annealing at 400 °C.

To achieve an EOT of 0.5 nm, gate dielectrics must have tolerance against interfacial layer formation during the heating processes after the fabrication of the gate stack. Therefore, the behavior of interfacial layer formation during heating processes due to the oxygen in the LaAlO_3_ film was examined by performing annealing in vacuum conditions (~4 × 10^−7^ Torr) for LaAlO_3_ films deposited at RT (hereinafter called “RT-LAO”) and at 700 °C (hereinafter called “HT-LAO”). Figure 8 (a), (b) respectively show Si 2s XPS spectra for RT-LAO and HT-LAO specimens with vacuum annealing temperatures ranging from 400 °C to 600 °C. TEM images after annealing at 400 °C are shown in the insets.

In the as-deposited state, no peak derived from an interfacial layer could be observed on either film, indicating that a direct LAO/Si structure was achieved, irrespective of the deposition temperature. Interfacial layer formation, which is judged to occur based on the growth of the oxide peaks in Si 2s (151~154 eV), is clearly observed in the RT- LAO specimens after vacuum annealing. In contrast, the Si 2s spectra for the HT-LAO specimens are almost unchanged by the annealing; indicating that direct contact of LaAlO_3_/Si is maintained up to 600 °C.

**Figure 9:**
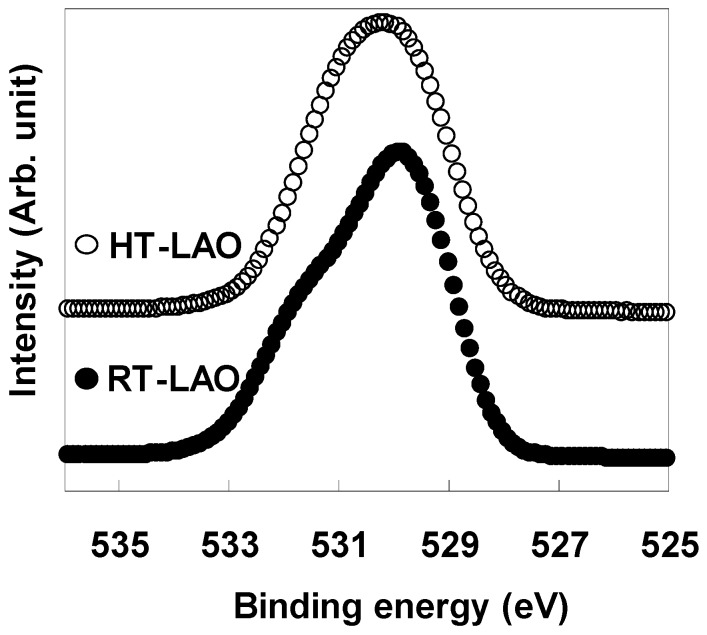
O 1s spectra for as-deposited RT-LAO and HT-LAO.

To investigate the origin of the difference in the interfacial layer formation behavior for the two deposition temperatures, the bonding states of the oxygen atoms in the as-deposited films were investigated by XPS. Figure 9 shows the O 1s spectra for the as-deposited RT-LAO and HT-LAO films. In the as-deposited HT-LAO film, a symmetric peak was observed at the same binding energy as in an LaAlO_3_ single crystal. This indicates that a microscopically homogeneous film structure is reached in the HT-LAO film. On the other hand, in the as-deposited RT-LAO film, an asymmetrical spectrum was obtained, indicating that multiple bonding states of oxygen exist in the film. This inhomogeneity in the bonding in the RT-LAO film is thought to be closely related to interfacial layer formation during vacuum annealing. The peak at the higher binding energy in the as-deposited RT-LAO may be attributable to an H_2_O or –OH group, which could be responsible for the interfacial layer formation during vacuum annealing.

**Figure 10:**
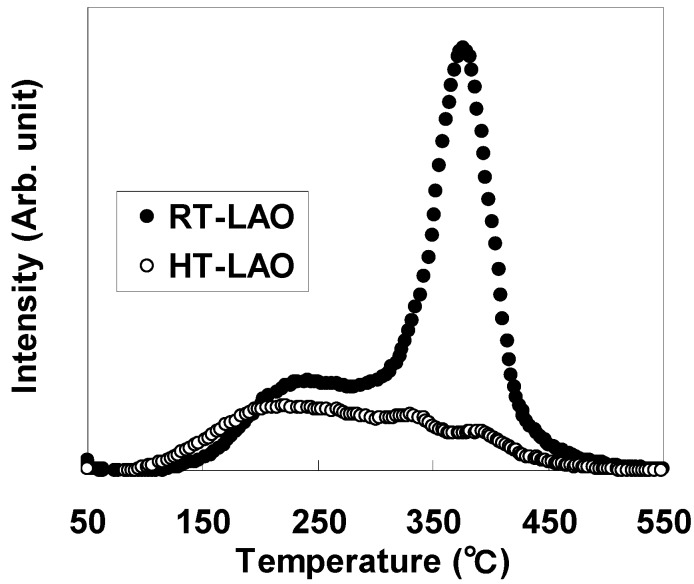
TDS spectra for mass 18 (H_2_O) for RT-LAO and HT-LAO.

For further clarification of this phenomenon, the desorbed components from LaAlO_3_ films during annealing were analyzed by TDS. Figure 10 shows the TDS spectra for mass 18 (H_2_O) for RT-LAO and HT-LAO. The two spectra differ significantly from each other. The peaks at around 200°C, which were similar in both spectra, were due to the surface adsorbate. The peaks at around 400°C, which were very different in the two spectra, were attributable to a component from the film interior. The area under the peak at 400 °C was more than 10 times greater for RT-LAO than for HT-LAO. The spectra for mass 17 (H_2_O) (not shown) were similar to those for mass 18 (H_2_O). These results suggest that RT-LAO contains a large amount of the OH group and H_2_O, which could diffuse into the film during annealing and cause interfacial layer formation. The HT deposition process can thus suppress the incorporation of these oxygen-related components during deposition and inhibit subsequent interfacial layer formation during annealing.

## 3. Interfacial Strain Induced by Direct Bonding of LaAlO_3_ Film to Si Substrate

Although an interface with direct bonding of a high-*k* dielectric to Si has generally been considered to have significantly different physical and electrical properties from the conventional SiO_2_/Si interface, the direct high-*k*/Si interface has not yet been characterized fully. In this section, the lattice strain at the interface between the gate dielectric and the Si substrate is examined, because it is an important parameter that directly influences the carrier mobility in MOSFET devices. The interface lattice strain, which depends on the interface structure, was investigated by comparing specimens with direct bonding of LaAlO_3_ to Si with specimens consisting of a stack with an interfacial layer (hereinafter referred to as IL) [21].

LaAlO_3_ films were deposited on HF-last Si (100) by the PLD method using a KrF excimer laser at a substrate temperature of 600 °C. One of the specimens was then annealed in an oxygen ambient at 600 °C for 30 min to generate an IL.

The elemental depth profile measurements and strain measurements were performed by the ion channeling technique using high-resolution Rutherford backscattering spectroscopy (HRBS). The details of HRBS are described elsewhere [22]. An He^+^ ion beam with an energy of 450 keV was aligned along the Si [111] direction, while the energy of the scattered He^+^ ions was analyzed by a magnetic spectrometer with a scattering angle of 50°. RBS angular scan measurements across the [111] direction were also performed in steps of 0.2° in order to evaluate the strain of Si near the interface.

### 3.1. LaAlO_3_/Si Structure

**Figure 11:**
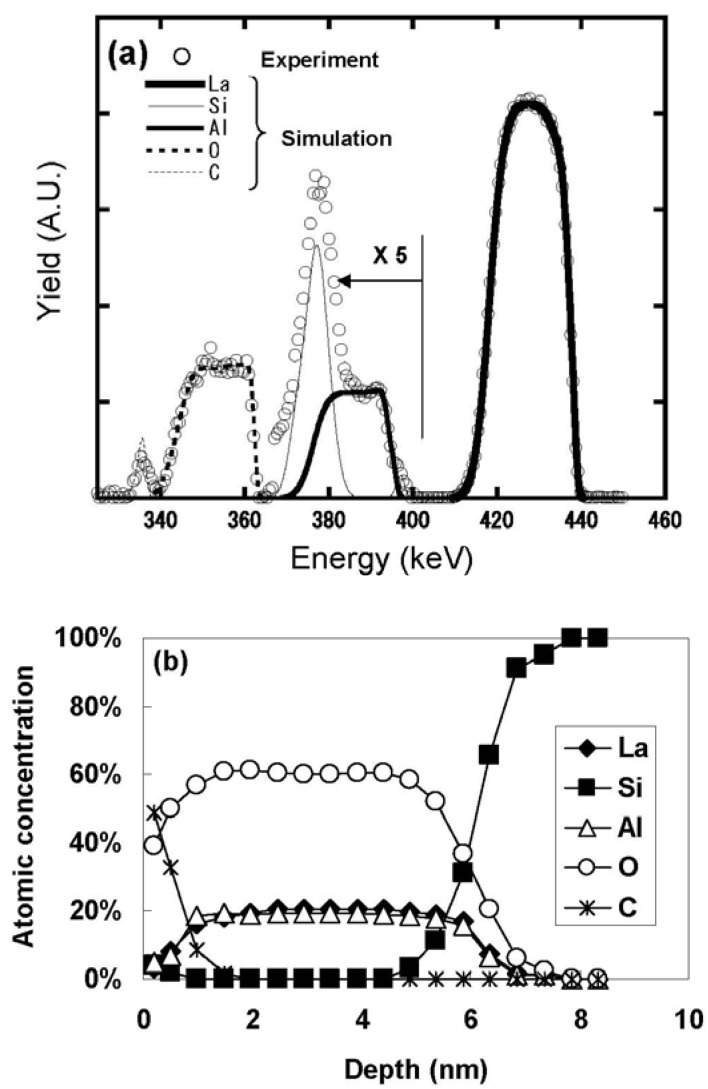
(**a**) HRBS spectrum for the as-deposited specimen; (**b**) Elemental depth profiles for the as-deposited specimen.

**Figure 12:**
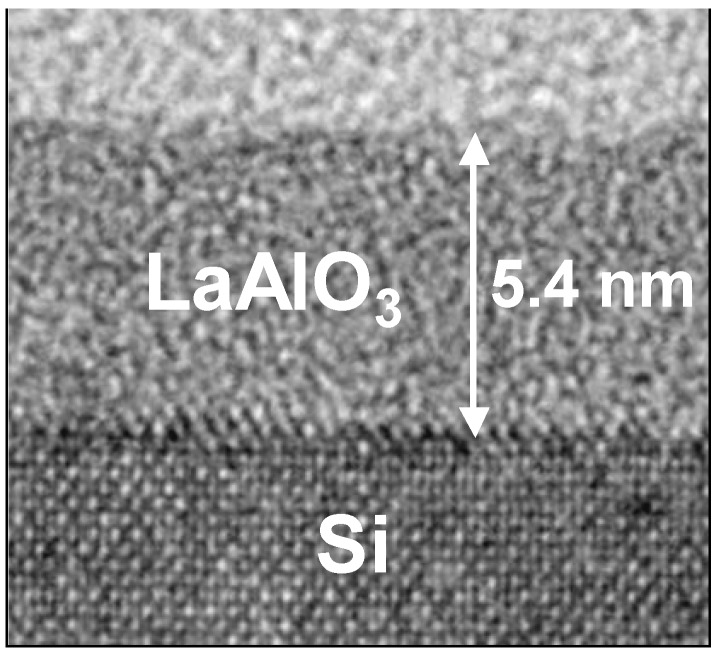
Cross-sectional TEM image of the as-deposited specimen.

Figure 11 (a) shows the HRBS spectrum for the as-deposited specimen, while Figure 11(b) shows the elemental depth profiles obtained by fitting the simulation model to the spectrum. Figure 11(b) indicates that the atomic ratio of La:Al:O in the LaAlO_3_ film is approximately 1:1:3. The sharp change of the elemental depth profiles at the interface with Si suggests that the LaAlO_3_ film was deposited directly on the Si substrate, without any IL. To confirm the absence of the IL, this stack was analyzed using TEM. Figure 12 shows a cross-sectional TEM image of the specimen whose HRBS spectrum is shown in Figure 11. No contrast difference is observed above the Si substrate, indicating the absence of an IL and direct bonding of LaAlO_3_ to Si.

**Figure 13:**
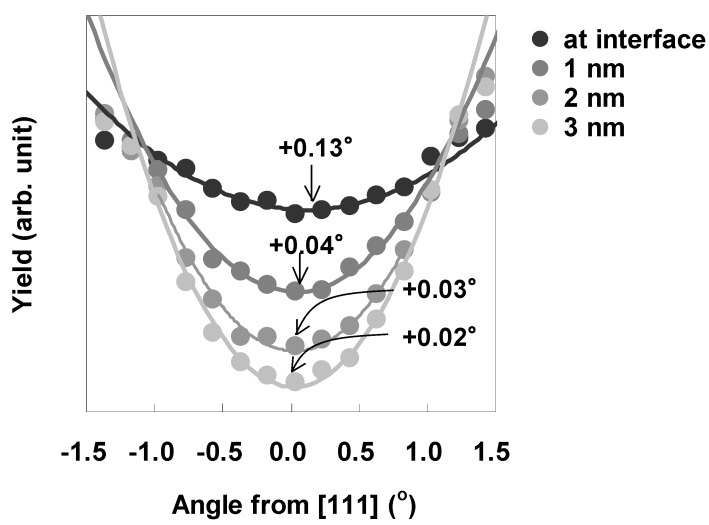
Scattering yield from Si at various depths from the interface as a function of the incident angle relative to the [111] direction for the as-deposited specimen.

**Figure 14:**
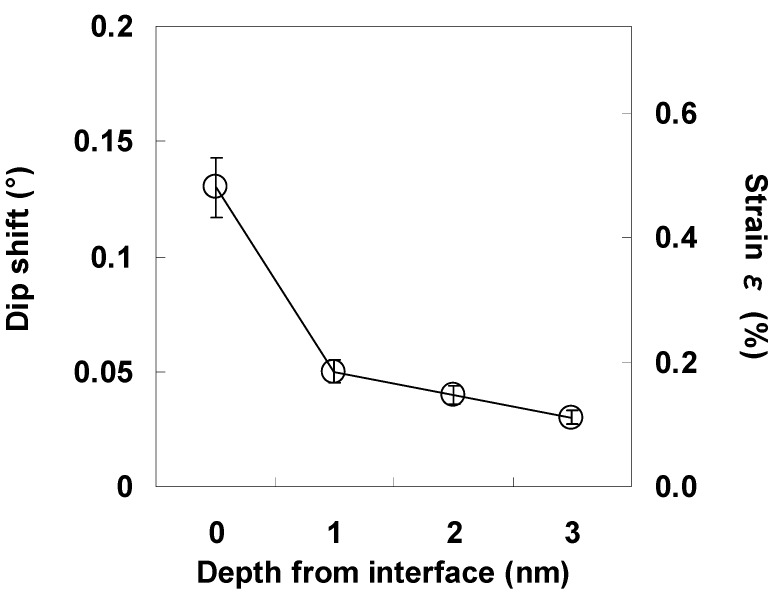
Depth dependence of the strain at the LaAlO_3_/Si interface.

Angular scan measurements were then performed to characterize the lattice strain of the Si bonded directly to LaAlO_3_. Figure 13 shows the scattering yield from Si at various depths from the interface as a function of the incident angle relative to the [111] direction, where a positive angle indicates inclination toward the surface and a negative angle indicates inclination toward the normal to the surface. Here, the interface is defined as the depth where the Si concentration is 85% in Figure 11(b) in order to eliminate as much of the interference of the adjacent Al signal as possible. The solid lines in the figure represent fitted quadratic functions. The minimum value of the fitted quadratic function at each depth was defined as the dip position of the curve. As can be seen clearly in Figure 13, the dip position shifts away from [111] in the positive direction as the distance from the interface decreases. The direction of this shift corresponds to the horizontal tensile strain in Si. Assuming that this tensile strain exists only in the horizontal direction, the magnitude of the strain ε can be approximated by Equation (2) below [23,24,25].

(2) $$\varepsilon =\frac{2\Delta \theta }{\sin2\theta }$$

where θ is the incident angle along the [111] channel and Δθ is the angular shift relative to the [111] direction. The depth profile of the strain estimated using equation (2) is shown in Figure 14. The strain at the interface can be seen to be as large as 0.5%, and decreases rapidly in the first nanometer from the interface, reaching a value of 0.2% at 1 nm.

### 3.2. LaAlO_3_/SiO_2_(IL)/Si Structure

**Figure 15:**
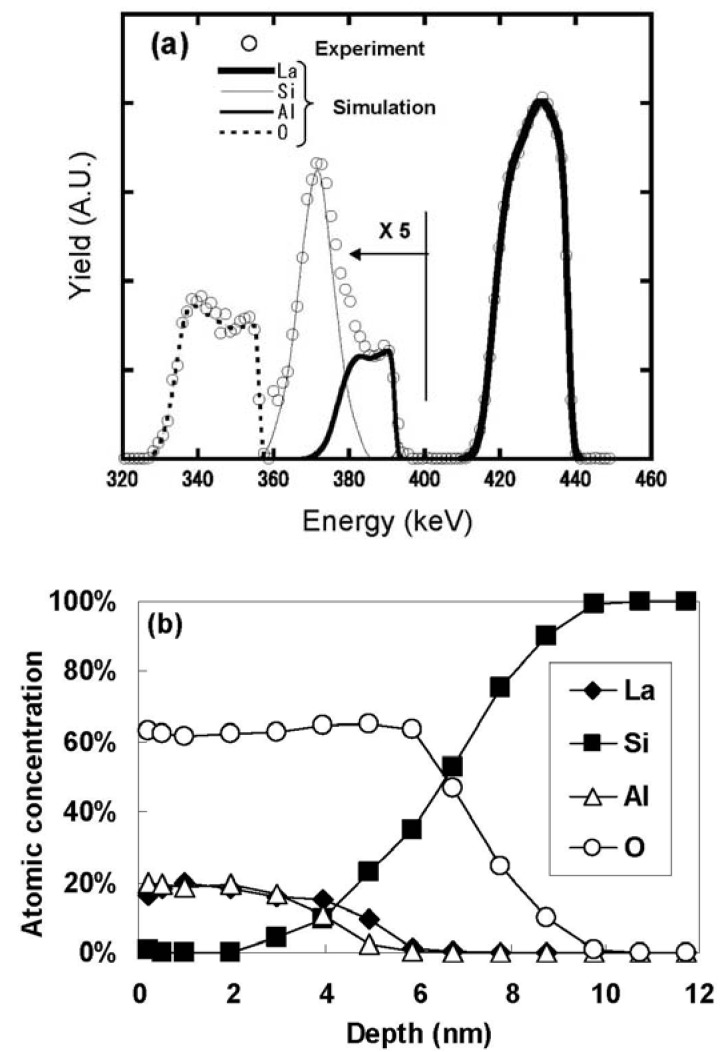
(**a**) HRBS spectrum for the specimen annealed in an oxygen ambient; (**b**) Elemental depth profiles for the specimen annealed in an oxygen ambient.

To determine whether the above results are unique to the direct interface of LaAlO_3_/Si, the same evaluation was performed for a specimen in which an IL was intentionally formed by annealing in an oxygen ambient. Figure 15(a) shows the HRBS spectrum for the specimen annealed in an oxygen ambient, while Figure 15(b) shows the elemental depth profiles for the specimen. In contrast with Figure 11(b), an IL composed of Si and O is clearly seen in the depth range of 6 to 8 nm. Note that the layer composed of La, Al, and O in the depth range of 4 nm from the surface kept the atomic ratio (La:Al:O = 1:1:3 ) even after oxygen annealing.

**Figure 16:**
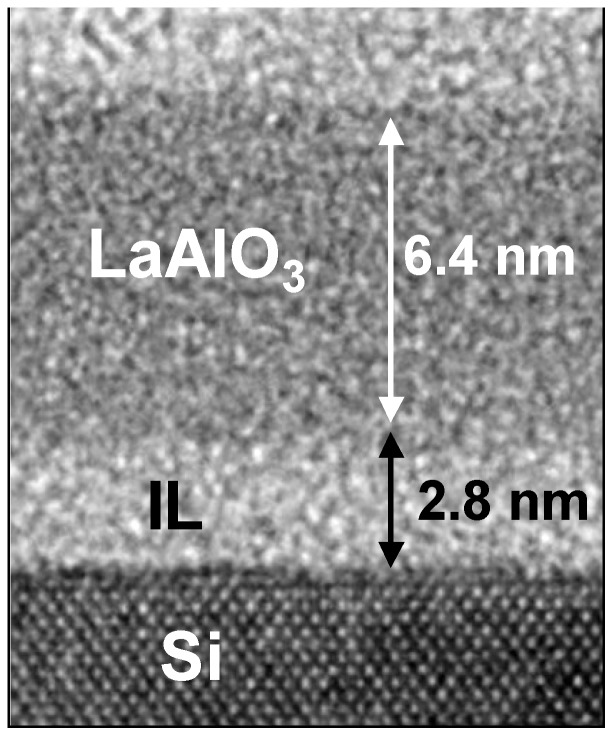
Cross-sectional TEM image of the specimen annealed in an oxygen ambient.

**Figure 17:**
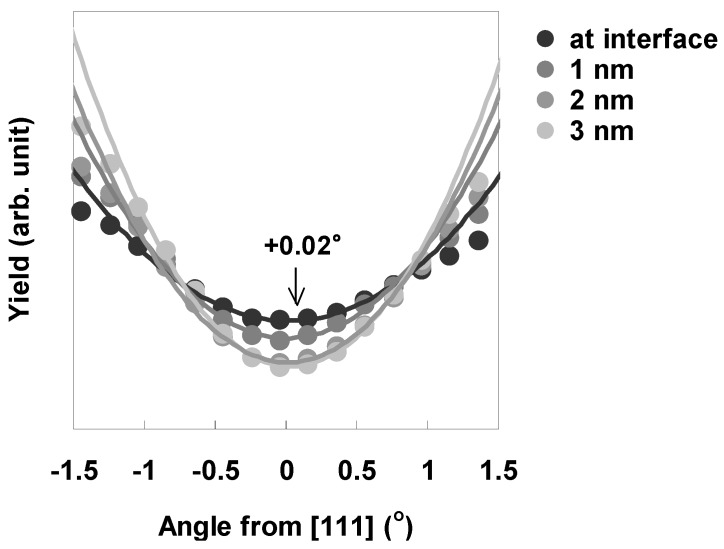
Scattering yield from Si at various depths from the interface as a function of the incident angle relative to the [111] direction for the specimen annealed in an oxygen ambient.

**Figure 18:**
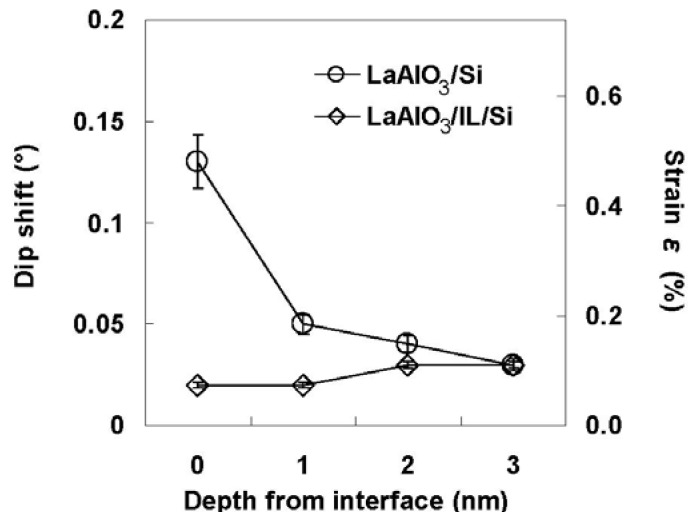
Depth dependence of the strain at the SiO_2_ (IL)/Si interface for the specimen annealed in an oxygen ambient. The data for the as-deposited specimen is shown again for comparison.

The TEM image shown in Figure 16 makes the stack structure of LaAlO_3_ and the IL more obvious. The relatively bright layer with a thickness of about 2.8 nm is the IL (SiO_2_). Angular scan measurements were also performed for the same annealed specimen (Figure 17). As can be seen in Figure 17, the depth dependence of the dip position is very small, and the shift of the dip from the [111] direction at the interface is as small as 0.02°. This result differs significantly from that in Figure 13 for the as-deposited specimen with direct bonding of LaAlO_3_ film to Si substrate. Figure 18 shows the depth profile of the strain for the annealed specimen. The figure also includes the data for the as-deposited specimen for comparison. The difference between the two specimens in Figure 18 is significant in the region from the interface to 1 nm, showing that large strain is characteristic of direct bonding of LaAlO_3_ film to Si.

These results suggest that the dielectric material in contact with the Si determines the magnitude of the strain in Si. The results of this study could therefore lead to novel strain engineering techniques that use gate dielectrics for higher channel mobility.

## 4. Stability of the Effective Work Function for La-Based High-*k* Materials

In this section, the stability of the effective work functions (φeff) for p-metals on La-based high-*k* materials is studied in detail for various annealing ambients and gate dielectric structures, because it has been widely reported that φeff for p-metals (such as Pt) on Hf-based high-*k* materials depends strongly on the annealing ambient [26,27,28]. The factors and the interfaces responsible for the variation of φeff will be discussed [29].

La_2_Hf_2_O_7_ (LHO) was selected as typical La-based high-*k* materials for the comparison with LaAlO_3_ (LAO). It is reported that LHO has been also grown directly on Si [30]. LAO and LHO films were deposited on a 5-nm-thick thermal oxide layer covering 3-inch p-Si wafers or HF-treated p-Si wafers by PLD using a KrF excimer laser. LaAlO_3_ single crystal pellets were used as the deposition target for LAO, while La_2_Hf_2_O_7_ sintered pellets were used as the deposition target for LHO.

**Figure 19:**
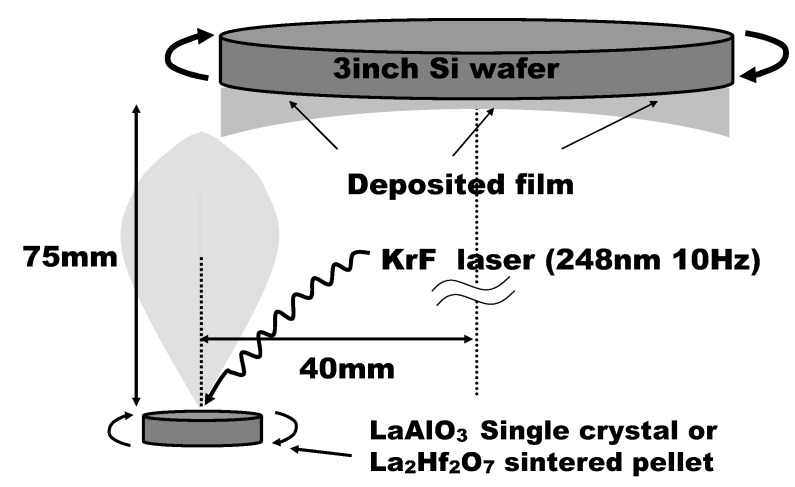
Schematic representation of the PLD system used in the experiments. The film thickness increases with the distance from the center of the wafer.

**Figure 20:**
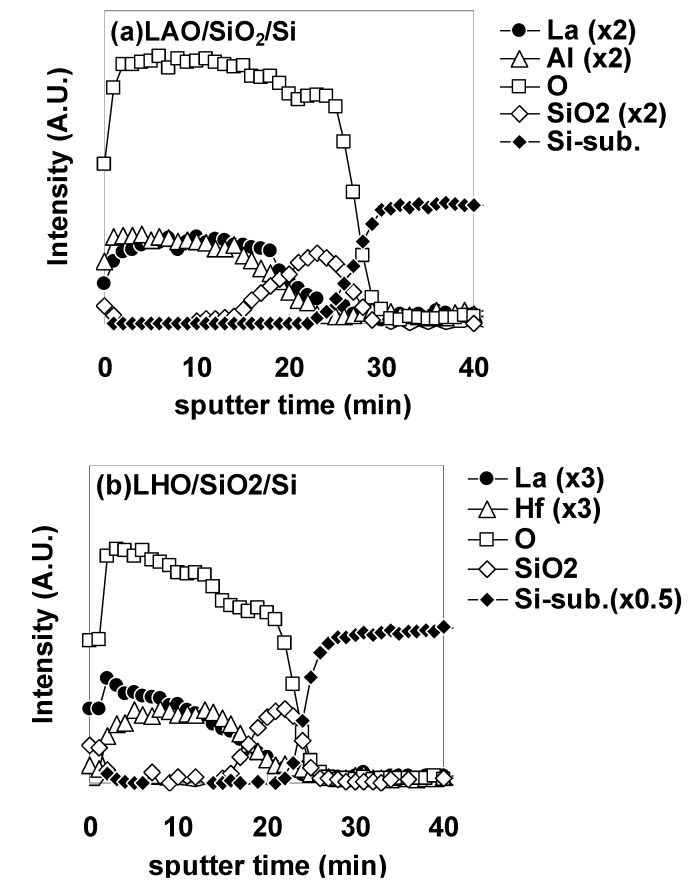
Typical depth profiles of the (**a**) LAO/SiO_2_/Si and (**b**) La_2_Hf_2_O_7_ (LHO)/SiO_2_/Si stacks. The detected Auger electron kinetic energies were selected so as to avoid overlapping of peaks. Target-factor analysis (TFA) was performed for the Si peaks in order to separate the composite peaks into Si oxide (SiO_2_) and Si metal (Si-substrate).

Figure 19 shows a schematic representation of the PLD system used in the experiments. As can be seen in the figure, deposition targets were set below the circumference of the wafer to deposit a film whose thickness increases from the center of the wafer to the circumference, enabling a gradual change of EOT values for accurate estimation of φeff. Pt was selected as a typical p-metal material. Pt films were deposited on LAO and LHO by e-beam evaporation through shadow masks to define the capacitor area, and MIS capacitors were fabricated. After the deposition of Pt, forming gas (H_2_/N_2_ = 10%) annealing (FGA) was performed at 450 °C for 30 min. Some specimens were additionally annealed in an O_2_ ambient, N_2_ ambient, or Ar ambient at 400 °C for 30 min after FGA. C-V measurements were carried out for the MIS capacitors. To estimate the elemental depth profile of each dielectric stack, Auger electron spectroscopy (AES) measurements were performed. Figure 20 shows the typical depth profiles of the (a) LAO/SiO_2_/Si and (b) LHO/SiO_2_/Si stacks. Target-factor analysis (TFA) was performed for the Si peaks in order to separate the composite peaks into Si oxide (SiO_2_) and Si metal (Si-substrate). As can be seen in Figure 20, the underlying SiO_2_ layer is clearly detected for both LAO and LHO.

### 4.1. Differences in the Annealing-Ambient-Dependence of φeff for Pt/LAO/SiO_2_/Si and Pt/LHO/SiO_2_/Si

To investigate whether or not the changes in φeff observed after FGA only and after additional O_2_ annealing in Hf-based high-*k* materials [26,27,28] also occur for La-based high-*k* materials, the C-V characteristics were examined after FGA only and after additional O_2_ annealing. Measurements were performed for gate stacks with an SiO_2_ interfacial layer (as in most of the reports on Hf-based high-*k* materials).

**Figure 21:**
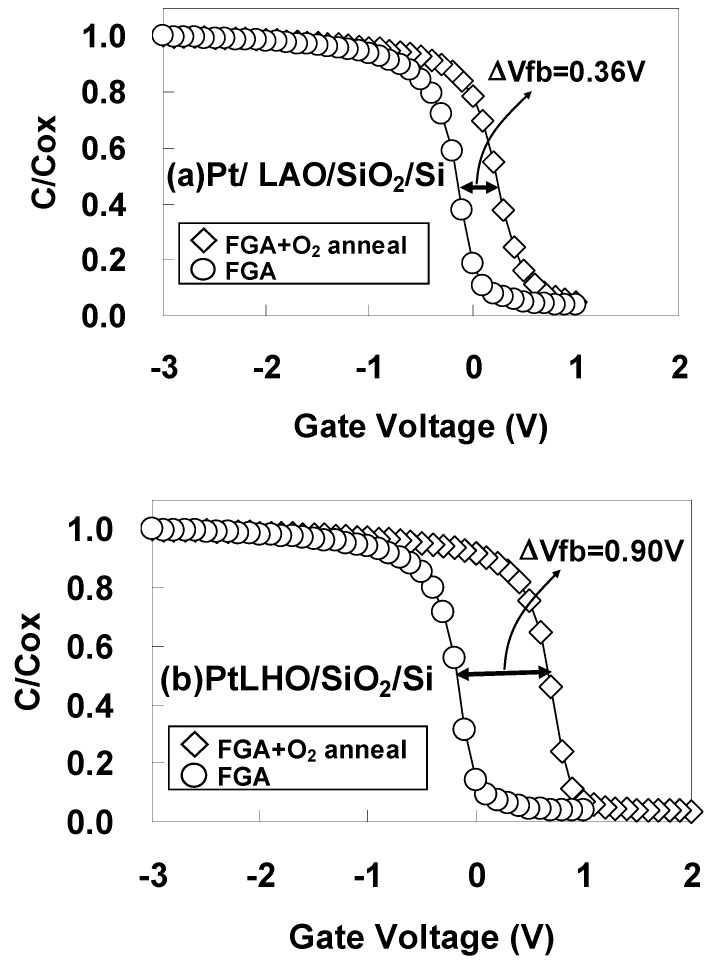
C-V curves for the highest capacitances for (**a**) Pt/LAO/SiO_2_/Si and (**b**) Pt/LHO/SiO_2_/Si MOS capacitors after forming gas annealing (FGA) only and after additional O_2_ annealing.

Figure 21 shows the C-V curves for the highest capacitances (as a typical example) for (a) Pt/LAO/SiO_2_/Si and (b) Pt/LHO/SiO_2_/Si MOS capacitors after FGA only and after additional O_2_ annealing. As can be seen in Figure 21, well-behaved C-V curves were obtained in all the cases, but there was a large difference in the annealing ambient dependences of these capacitors. While the difference in the Vfb values after FGA only and after additional O_2_ annealing (ΔVfb) did not exceed 0.36 eV for the Pt/LAO/SiO_2_/Si stack, a significantly larger Vfb difference of 0.90 eV was observed for the Pt/LHO/SiO_2_/Si stack.

**Figure 22:**
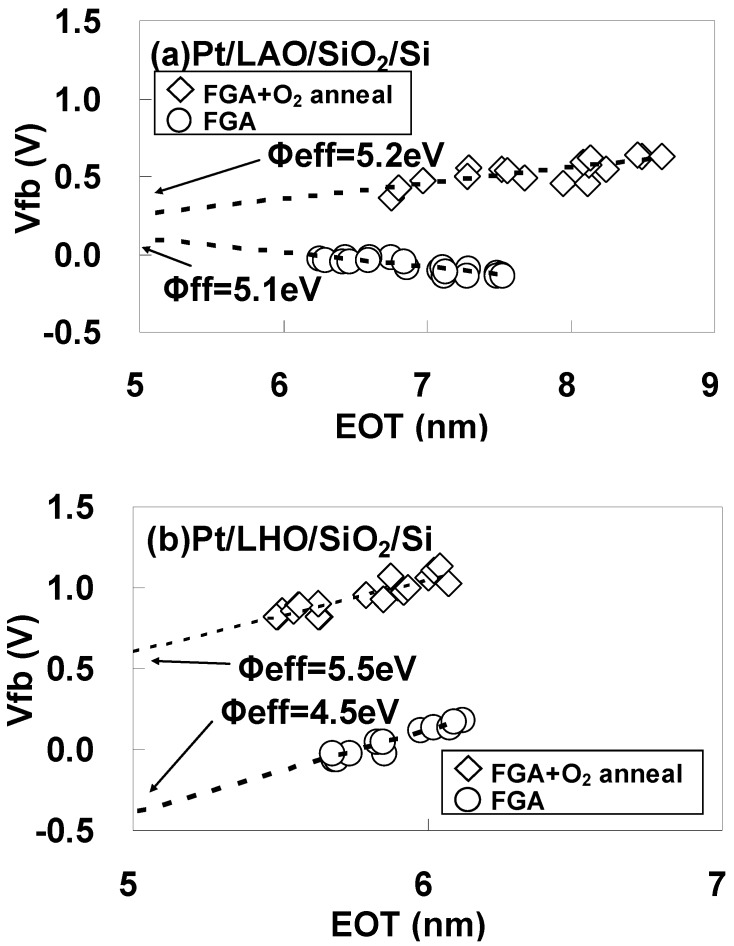
Dependence of Vfb on EOT after FGA only and after additional O_2_ annealing for (**a**) Pt/LAO/SiO_2_/Si and (**b**) Pt/LHO/SiO_2_/Si MOS capacitors. φeff was estimated from the point of intersection of the EOT = 5 nm vertical line with the extrapolated experimental data by assuming that a fixed charge was responsible for the local slope of the plot at the interface between the 5-nm SiO_2_ layer and LAO or LHO. This assumption is based on the experimental result that the dependence of Vfb on the SiO_2_ thickness was small for Pt/SiO_2_/Si capacitors (data not shown).

To estimate φeff accurately, the dependence of Vfb on EOT was obtained after FGA only and after additional O_2_ annealing for (a) Pt/LAO/SiO_2_/Si and (b) Pt/LHO/SiO_2_/Si MOS capacitors as shown in Figure 22. The estimated φeff values are also shown in the figures. As can be seen, the φeff behavior differs for these two kinds of stacks. In Figure 22(a), it can be seen that the Pt/LAO/SiO_2_/Si stack has a relatively high φeff, close to the vacuum work function of Pt (5.6 eV), even after FGA, and the φeff increase caused by the additional O_2_ annealing is small. However, the behavior for the Pt/LHO/SiO_2_/Si stack is quite different, as can be seen in Figure 22(b). The φeff for the Pt/LHO/SiO_2_/Si stack after FGA is as low as 4.5 eV, which is much lower than the values for the Pt/LAO/SiO_2_/Si stack and the vacuum work function of Pt. Thus, the lowering of φeff in the LHO/SiO_2_/Si stack by the FGA process, which was reported in reference [31], was confirmed. It was, however, newly found that additional O_2_ annealing enables recovery of φeff to a value close to the vacuum work function of Pt, as can be seen in Figure 22(b). This anomalous behavior of φeff in the LHO/SiO_2_/Si stack is thought to be caused by differences in the dipole contribution to φeff depending on the annealing ambient. The origin and locations of these dipoles are discussed below.

### 4.2. Physical Origin of the Annealing-Ambient-Dependence of φeff for the Pt/LHO/SiO_2_/Si Stack

The oxygen atoms themselves and the thermal process during O_2_ annealing could both be possible contributing factors to the dramatic recovery of φeff seen in Figure 22(b) as a result of additional O_2_ annealing. In order to distinguish between these two factors, additional N_2_ annealing and additional Ar annealing were performed instead of additional O_2_ annealing for Pt/LHO/SiO_2_/Si MOS capacitors under the same temperature and time conditions as for O_2_ annealing (450 °C, 30 min).

**Figure 23:**
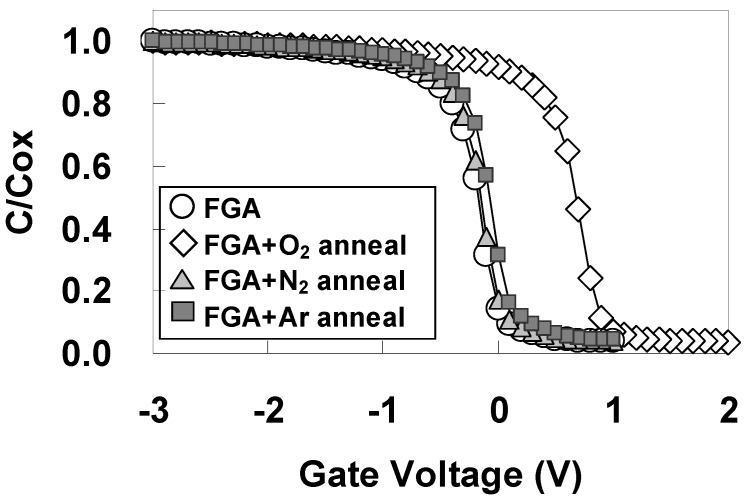
C-V curves for the highest capacitances after additional N_2_ and Ar annealing. The curves after only FGA and after additional O_2_ annealing are shown again for comparison.

**Figure 24:**
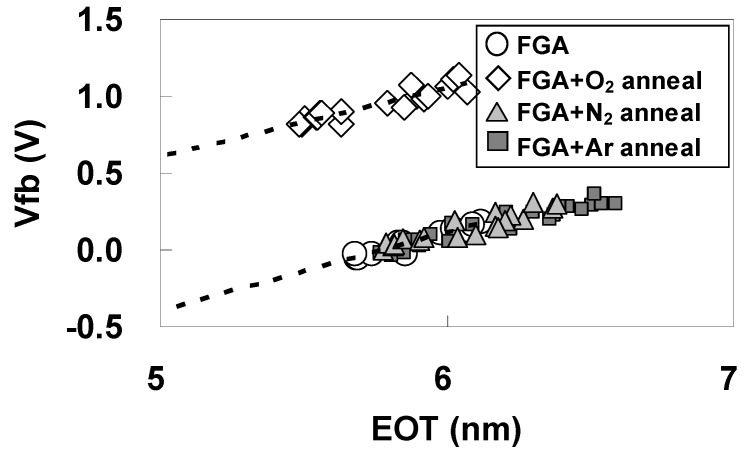
Dependence of Vfb on EOT after additional N_2_ and Ar annealing. The curves after only FGA and after additional O_2_ annealing are shown again for comparison.

Figure 23 shows the C-V curves for the highest capacitances (as a typical example) after additional N_2_ and Ar annealing for Pt/LHO/SiO_2_/Si MOS capacitor, with the curves after FGA only and after additional O_2_ annealing shown again for comparison. As can be seen in Figure 23, in contrast to the case of additional O_2_ annealing, neither additional N_2_ annealing nor additional Ar annealing has much effect on the C-V characteristics, including Vfb. The EOT dependences of Vfb after additional N_2_ annealing and additional Ar annealing for Pt/LHO/SiO_2_/Si MOS capacitors are also similar to that after FGA only as can be seen in Figure 24, indicating that φeff is not affected by either N_2_ annealing or Ar annealing. Thus, the thermal process itself does not affect φeff. These results indicate that oxygen-vacancy-related dipoles caused by FGA are responsible for lowering of φeff, and oxygen annealing inactivates these dipoles through re-oxidation.

It should be noted that while the φeff in the Pt/LHO/SiO_2_/Si stack changes with additional O_2_ annealing, the positive slopes of the EOT dependences of Vfb are unaffected by the variation in annealing ambient as can be seen in Figure 22(b) and Figure 24. Oxygen vacancies generally produce positive charges, resulting in a negative slope of the EOT dependences of Vfb. Considering this, it is thought that the positive slopes of the EOT dependences of Vfb are the result of the existence of large amounts of negative fixed charges in the Pt/LHO/SiO_2_/Si stack, and that these negative charges are unaffected by the use of different annealing ambients. On the other hand, the positive charges produced by oxygen vacancies are unlikely to affect the slope, because they are thought to be negligible in comparison with negative fixed charges. Since this type of unstable φeff phenomenon was not observed in the Pt/LAO/SiO_2_ stack, LAO is considered to be less susceptible to oxygen-vacancy formation than LHO.

**Figure 25:**
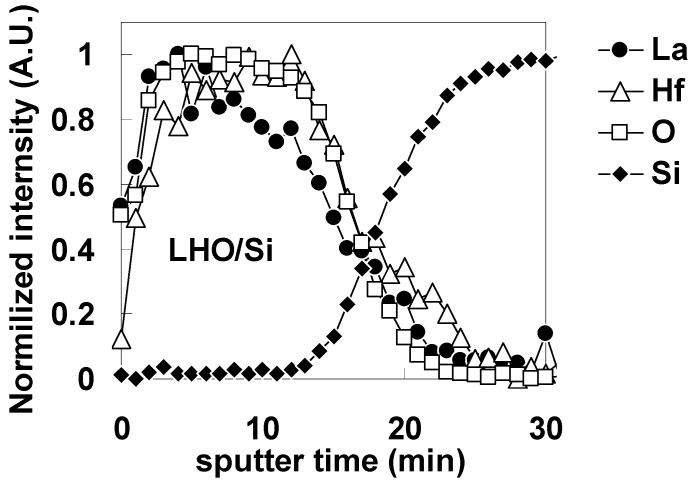
Auger electron spectroscopy (AES) depth profile for the LHO/Si stack. The vertical axis is normalized to show the sharpness of the interface more clearly.

Next, in order to determine the locations of the oxygen-vacancy-related dipoles in the Pt/LHO/SiO_2_/Si stack, a stack without the SiO_2_ interfacial layer (Pt/LHO/Si) was fabricated by LHO deposition on an HF-treated Si surface, and the same experiments as for the stack with the SiO_2_ interfacial layer were performed. Figure 25 shows the AES depth profile for the LHO/Si stack. As can be seen in Figure 25, a sharp LHO/Si interface was obtained. TFA revealed that the Si peak was composed of a single component, only Si metal (Si substrate), indicating a very thin (if any) interfacial layer in the stack.

**Figure 26:**
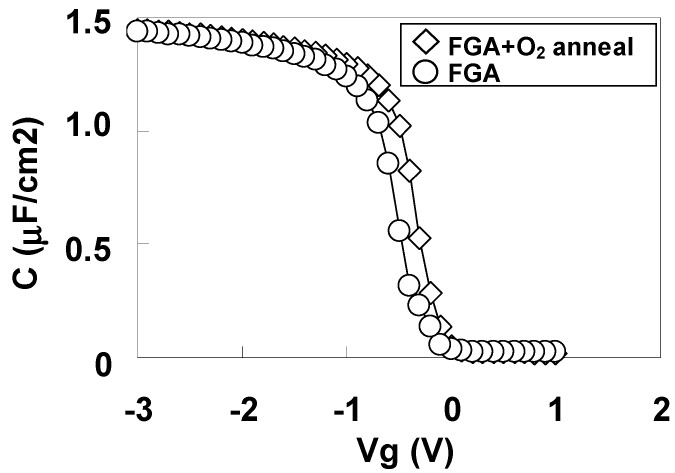
C-V curves with the highest capacitances for Pt/LAO/Si MOS capacitors after FGA only and after additional O_2_ annealing.

Figure 26 shows the C-V curves for the highest capacitances (as a typical example) for Pt/LHO/Si MOS capacitors after FGA only and after additional O_2_ annealing. In contrast to the result for the stack with the SiO_2_ layer (Figure 21(b)), the Vfb difference between these curves is small.

**Figure 27:**
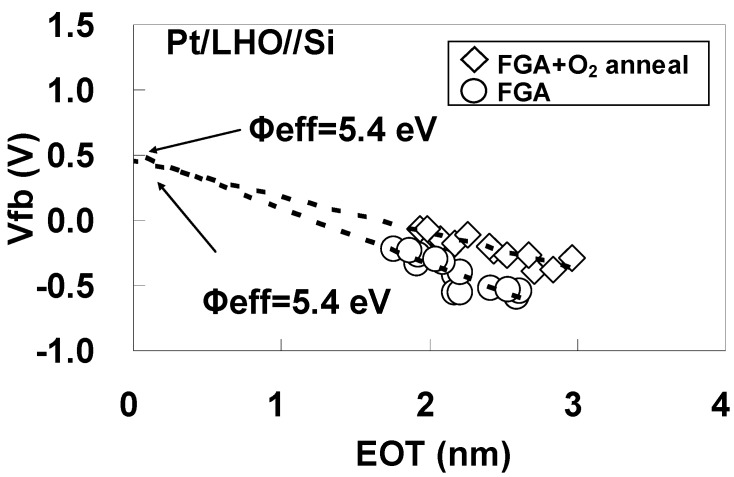
Dependence of Vfb on EOT for the specimens after FGA only and after additional O_2_ annealing. φeff was estimated from the point of intersection of the Y axis with the extrapolated experimental data by assuming that a fixed charge was responsible for the local slope of the plot at the interface between LHO and the Si substrate.

To estimate φeff accurately, the dependence of Vfb on EOT was obtained after FGA only and after additional O_2_ annealing for Pt/LHO/Si MOS capacitors as shown in Figure 27. The estimated φeff values are also shown in the figure. The φeff values after FGA only and after additional O_2_ annealing were both 5.4 eV, which is close to the vacuum work function of Pt. This behavior is completely different from that of the stack with the SiO_2_ layer (Figure 22(b)).

The above results indicate that the LHO/Si and Pt/LHO interfaces do not include the oxygen-vacancy-related dipoles that are thought to be responsible for lowering φeff. Based on these findings, it can be concluded that the oxygen-vacancy-related dipoles observed in the Pt/LHO/SiO_2_/Si stack are located at the LHO/SiO_2_ interface. In addition, considering the φeff stability of LAO mentioned above, it is likely that the oxygen-vacancy-related dipoles observed in the Pt/LHO/SiO_2_/Si stack are caused by Hf atoms. Also, the reported φeff instability of Hf-based high-*k* materials, in which oxygen vacancies probably form as a result of FGA or high-temperature annealing at around 1000 °C, may have the same mechanism as that observed in the Pt/LHO/SiO_2_/Si stack in this study.

## 5. Impact of the Lanthanum Aluminate Composition on Vfb

In this section, the variation of the flat-band voltage (Vfb) behavior with the La/(La+Al) atomic ratio will be examined and the proposed guidelines for achieving a dual high-*k* gate stack structure using an La-Al-O dielectric system will be discussed. La oxide and Al oxide are promising materials for threshold voltage (Vth) tuning in high-*k* gate dielectrics [32,33,34]. Therefore, an La-Al-O ternary oxide system is considered to consist of materials applicable to a range of LSI process technologies now. However, the impacts of the La-Al-O composition on Vfb have not yet been investigated. We therefore carefully investigated the flat-band voltage (Vfb) behavior as a function of the La/(La+Al) atomic ratio [35].

**Figure 28:**
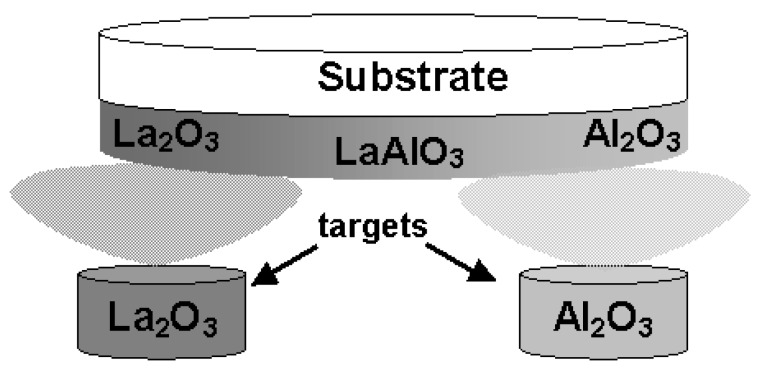
Schematic diagram of the positional relationships between the targets and substrate.

**Figure 29:**
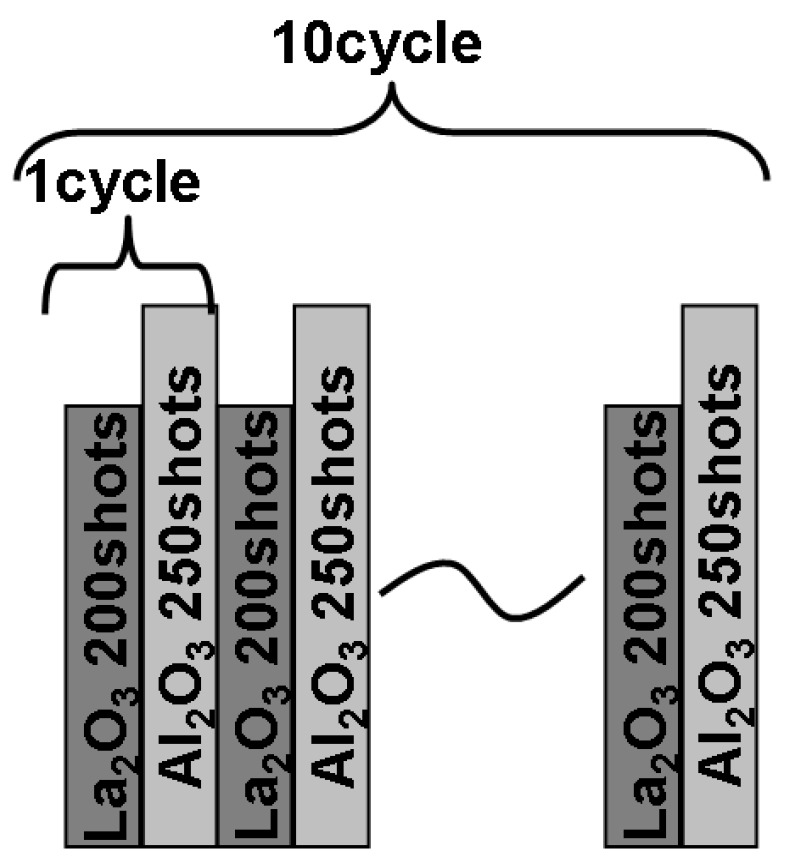
Laser irradiation sequence.

La-Al-O gate dielectric films were deposited on HF-treated p-Si(100) wafers at 600 °C by PLD. Sintered pellets of La_2_O_3_ and Al_2_O_3_ were used as deposition targets. These targets were set above each wafer edge as illustrated in Figure 28 in order to deposit a film whose composition changes gradually with position along a diameter of the wafer. In addition, film thickness also changed keeping constant La/(La + Al) along the direction perpendicular to the direction changing La/(La+Al). Figure 29 shows the laser irradiation sequence. Ten cycles of alternate laser irradiation (300 mJ, 10 Hz) were performed for the La_2_O_3_ and Al_2_O_3_ targets to achieve a uniform composition in the depth direction. Each cycle consisted of 200 shots for the La_2_O_3_ target and 250 shots for the Al_2_O_3_ target.

To estimate the atomic ratio of La/(La+Al) at different positions on the wafer, AES measurements were performed along the diameter of the wafer. After the fabrication of capacitors with Mo gates, forming gas (H_2_/N_2_ = 0.03) annealing was performed at 450 °C for 30 min. An Mo/SiO_2_/Si gate stack capacitor was prepared as a reference. Gate dielectric stacks in which La-Al-O film was deposited on a 5-nm-thick (nominal thickness) thermal oxide (SiO_2_) interfacial layer were also fabricated to examine the contribution of the SiO_2_ interfacial layer to the Vfb shift [34]. In order to clarify the mechanism of the Vfb shift caused by the difference in composition, X-ray photoelectron spectroscopy (XPS) measurements were performed.

**Figure 30:**
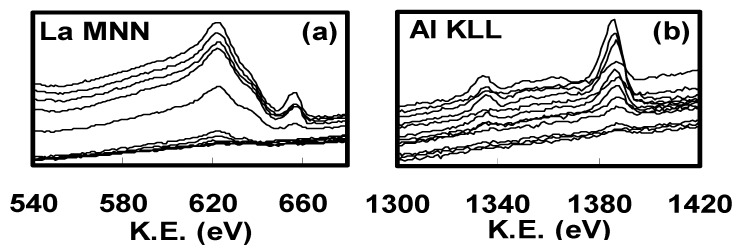
AES spectra along the diameter of the wafer for (**a**) La MNN and (**b**) Al KLL.

**Figure 31:**
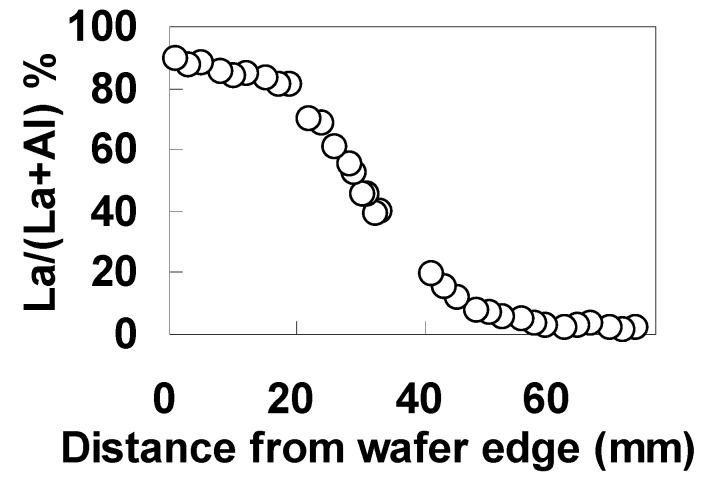
Dependence of La/(La+Al) on the position on the wafer.

Figure 30 shows the AES spectra of (a) La MNN and (b) Al KLL along the wafer diameter for the specimen without the underlying 5-nm SiO_2_ interfacial layer. Figure 31 shows the dependence of La/(La+Al) on the position on the wafer estimated from the intensities of AES spectra shown in Figure 30. Here, the atomic ratio was corrected using a standard specimen with La:Al:O = 1:1:3. As can be seen in Figure 31, the ratio of La/(La+Al) changed gradually along the diameter of the wafer, from 90% to close to 2%. Similar results to those in Figure 31 were also obtained for the specimen with the underlying 5-nm SiO_2_ layer.

**Figure 32:**
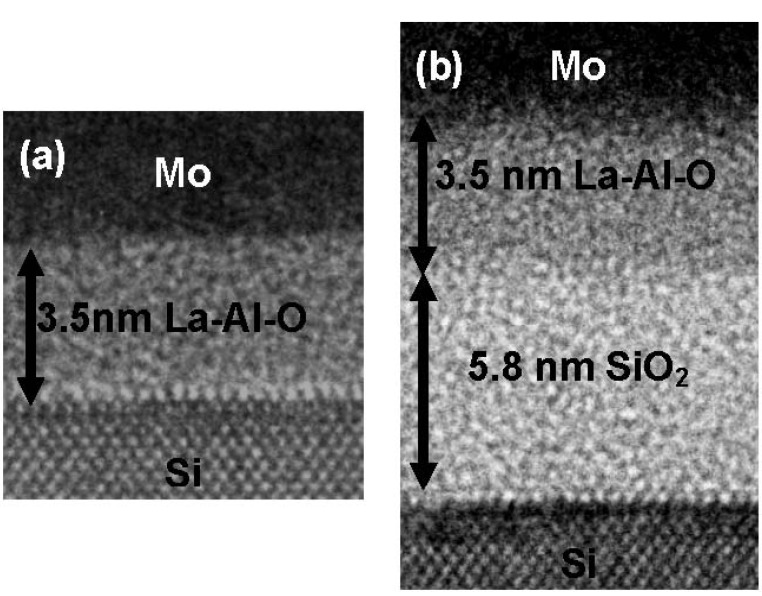
TEM images for (**a**) Mo/La-Al-O/Si and (**b**) Mo/La-Al-O/SiO_2_/Si gate stacks with La/(La+Al) = 50%.

Figure 32 shows TEM images for (a) Mo/La-Al-O/Si and (b) Mo/La-Al-O/SiO_2_/Si gate stacks in which La/(La+Al) = 50%. The thicknesses of the La-Al-O films ((a) and (b)) and the SiO_2_ layer (b) are shown in the images. The only difference between these images is the existence of the SiO_2_ layer between the La-Al-O film and the Si substrate in Figure 32(b). The contrast of the La-Al-O films is uniform in both cases, indicating that the alternately deposited ultrathin La_2_O_3_ layers and Al_2_O_3_ layers were well-mixed with each other during the deposition process at 600 °C.

**Figure 33:**
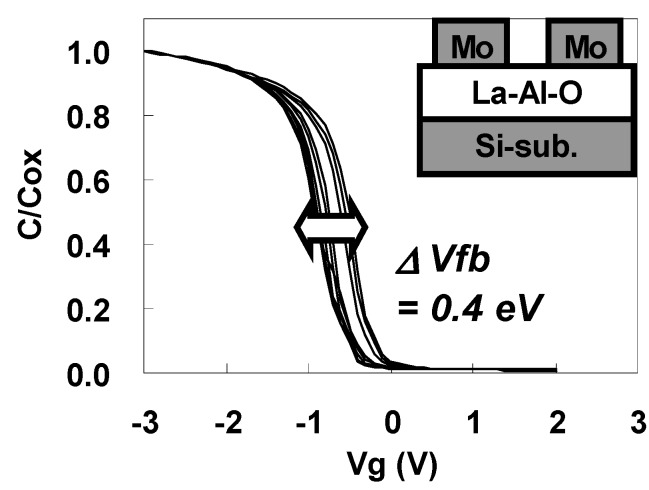
C-V curves for Mo/La-Al-O/Si gate stack capacitors along the wafer diameter over which the composition was changed.

Figure 33 shows the C-V curves for the Mo/La-Al-O/Si gate stack capacitors taken along the wafer diameter over which the composition was changed. Vfb is shifted in the positive direction as La/(La+Al) decreases and in the negative direction as La/(La+Al) increases. The maximum difference of Vfb (ΔVfb) between the obtained C-V curves shown in Figure 6 was 0.4 eV.

**Figure 34:**
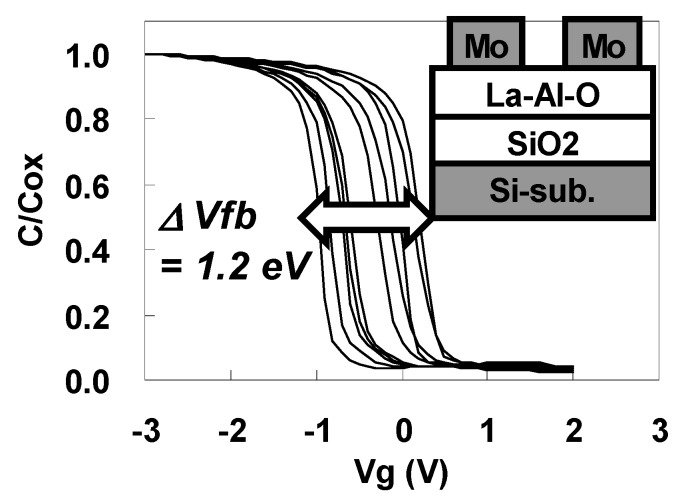
C-V curves for Mo/La-Al-O/SiO_2_/Si gate stack capacitors along the wafer diameter over which the composition was changed.

Figure 34 shows the C-V curves for the Mo/La-Al-O/SiO_2_/Si gate stack capacitors taken along the wafer diameter over which the composition was changed. As can be seen by comparing Figure 33 and Figure 34, insertion of the SiO_2_ changes the Vfb behavior dramatically. A large ΔVfb of 1.2 eV, greater than the bandgap energy of Si (1.12 eV), was observed for Mo/La-Al-O/SiO_2_/Si stacks. These results suggest that the La-Al-O/SiO_2_ interface makes a strong contribution to the Vfb shift.

**Figure 35:**
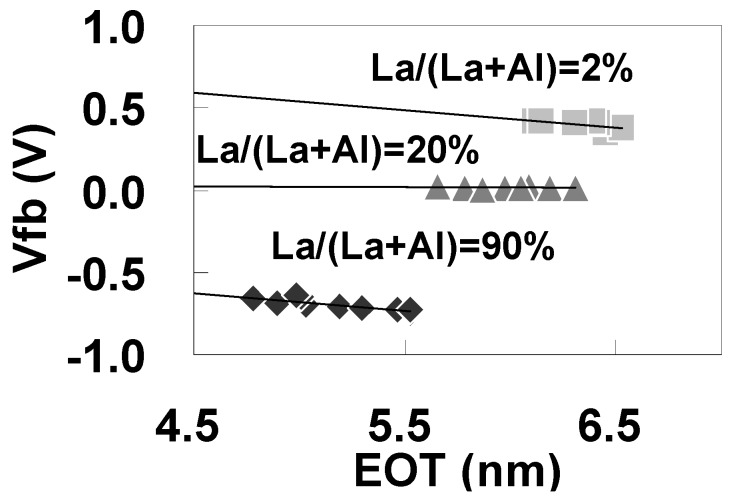
Dependence of Vfb on EOT for Mo/La-Al-O/SiO_2_/Si stacks with La/(La+Al) = 2%, 20%, and 90%. C-V measurements were performed in the direction perpendicular to the direction of La/(La+Al) variation.

It is well known that Vfb is affected by interfacial dipoles and fixed charges. To identify the factors causing the Vfb shift, the dependence of Vfb on the La-Al-O film thickness was investigated. Figure 35 shows the dependence of Vfb on EOT for Mo/La-Al-O/SiO_2_/Si stacks with La/(La+Al) = 2%, 20%, and 90%. No significant difference in the slopes is seen in Figure 35 for the different La/(La+Al) values, suggesting that there is no contribution of the fixed charges in the stacks to the change in Vfb with La/(La+Al). Thus, it can be concluded that dipoles at the La-Al-O/SiO_2_ interface are mainly responsible for the large ΔVfb observed in Figure 34. A similar relationship between EOT and Vfb was also observed for the Mo/La-Al-O/Si stacks.

**Figure 36:**
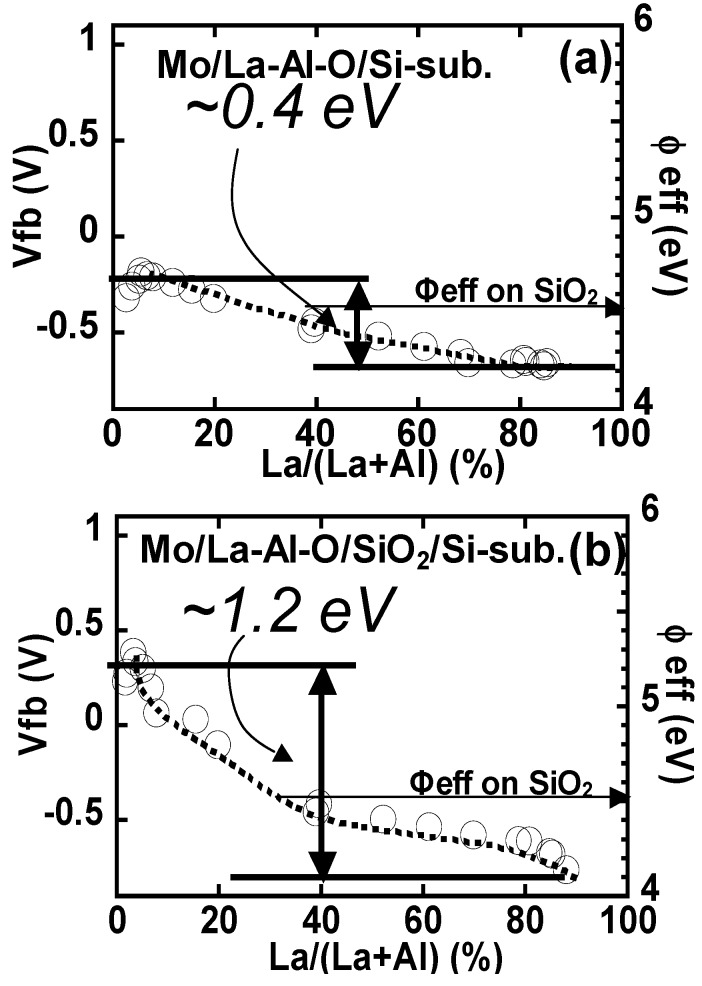
Plots of Vfb and φeff as functions of La/(La+Al) for (**a**) Mo/La-Al-O/Si and (**b**) Mo/La-Al-O/SiO_2_/Si gate stack capacitors.

Figure 36 shows the plots of Vfb and the effective work function (φeff) as functions of La/(La+Al) for (a) Mo/La-Al-O/Si and (b) Mo/La-Al-O/SiO_2_/Si gate stack capacitors. The same φeff as for the reference SiO_2_ film (4.54 eV; approximately equal to the intrinsic value) was obtained when La/(La+Al) was approximately 30%. This result suggests that interfacial dipoles with opposite orientations (due to the La and Al atoms) coexist at the La-Al-O/SiO_2_ interface, and the effects of these dipoles on Vfb cancel each other at an La/(La+Al) of approximately 30%. The dependence of Vfb on La/(La+Al) becomes weak above 30%, indicating that in La-Al-O systems it may be possible to use a wide La/(La+Al) range for Vth tuning of n-MISFETs.

**Figure 37:**
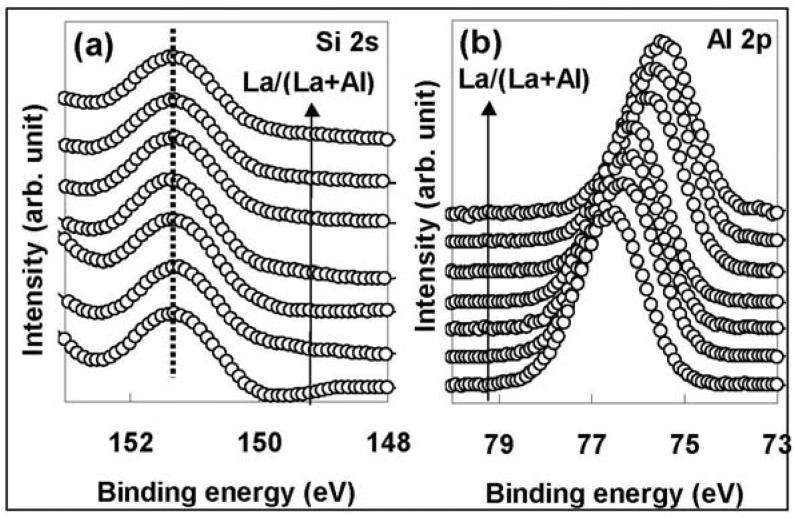
XPS spectra for (**a**) Si 2s from Si substrate and (**b**) Al 2p from La-Al-O film for La/(La+Al) values ranging from 2.8% to 75.4%.

The contribution of the dipoles to the change in Vfb with La/(La+Al) was also clarified by the dependence of the energy band alignment between the La-Al-O film and the Si substrate on La/(La+Al), which was investigated by measuring the energy difference between Al 2p for the La-Al-O film and Si 2s for the Si substrate using XPS. The absence of peak shifts caused by variation of the X-ray irradiation time during the XPS measurements was confirmed. Figure 37 shows the XPS spectra for (a) Si 2s from Si substrate and (b) Al 2p from La-Al-O film for La/(La+Al) values ranging from 2.8% to 75.4%, with the peak heights normalized to unity for comparison. As can seen in Figure 37(b), there is a clear negative binding energy shift of Al 2p relative to Si 2s with the increase of La/(La+Al), indicating that the valence band offset (ΔEv) decreases with the increase of La/(La+Al).

On the other hand, with regard to the stack without the SiO_2_ interfacial layer, a recent report indicates that the binding energy of Al 2p and ΔEv at the La-Al-O/Si interface show little dependence on La/(La+Al) at La/(La+Al) values of between 25% and 50% [36]. Therefore, it can be concluded that the La/(La+Al)-dependent voltage drop between the La-Al-O film and Si substrate occurs because of insertion of the SiO_2_ layer. This behavior is in good agreement with the dipole-induced Vfb shift mechanism referred to above.

**Figure 38:**
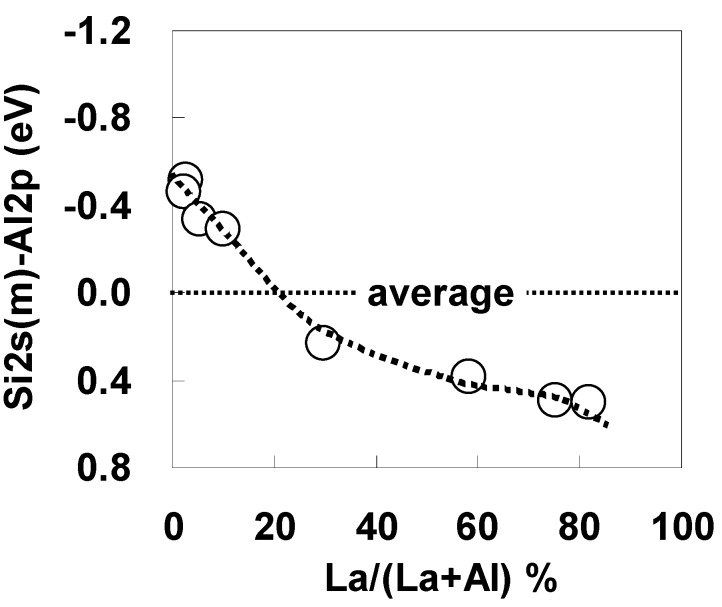
La/(La + Al) dependence of the energy difference between Al 2p and Si 2s. The vertical axis of the graph represents the energy difference as the divergence from the average value (average for all specimens).

Figure 38 shows the La/(La+Al) dependence of the energy difference between Al 2p and Si 2s. Note that the vertical axis of the graph represents the energy difference as the divergence from the average value. The behavior shown in Figure 38 is remarkably similar to that in Figure 36(b). This agreement indicates that there is no influence of carrier trapping during the voltage sweep for C-V measurements on the Vfb shift and the existence of dipoles in the stack.

**Figure 39:**
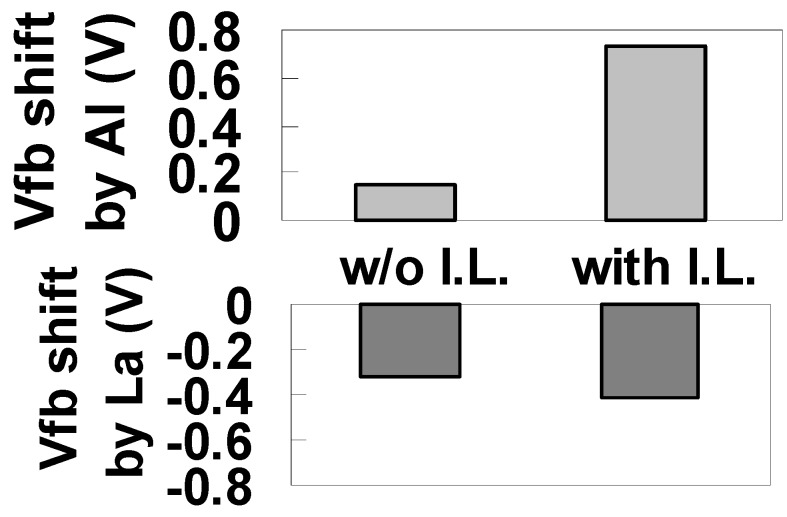
Bar graphs illustrating the maximum Vfb shift induced by (**a**) Al and (**b**) La relative to the intrinsic value for SiO_2_ film for the Mo/La-Al-O/SiO_2_/Si (with I.L.) and Mo/La-Al-O/Si (w/o I.L.) gate stacks.

Figure 39 illustrates the maximum ΔVfb induced by (a) Al and (b) La relative to the intrinsic value for SiO_2_ film for the Mo/La-Al-O/SiO_2_/Si and Mo/La-Al-O/Si gate stacks. Although the shift induced by Al without SiO_2_ was small, a large positive shift was induced by SiO_2_ insertion, indicating that SiO_2_ insertion is effective for Vth tuning of p-MISFETs. On the other hand, the negative shift induced by La without SiO_2_ was relatively large, and the increase in shift caused by the insertion of SiO_2_ was small.

Based on the findings above, we propose the following dipole model. Although an La-induced dipole forms at the interface with both Si and SiO_2_, the effect is slightly stronger at the SiO_2_ interface than at the Si interface. In addition, the effect of the Al-induced dipole becomes large when an SiO_2_ layer is inserted. Furthermore, comparison of cases with and without SiO_2_ in Figure 38 indicates that the moment induced at the SiO_2_ interface by Al is slightly larger than that induced by La. These results suggest that careful control of the interface layer is needed for Vth tuning of p-MISFETs with Al.

## 6. Gate-First TiN/LaAlO_3_ n-MOSFETs with Sulfur-Implanted Schottky Source/Drain Fabricated Using a Low-Temperature Process

As in the case of most high-*k* dielectrics on Si substrate, when a conventional gate-first process with high-temperature annealing at around 1000 °C for the activation of implants is applied to a gate stack with LaAlO_3_ film, the Si easily diffuses from the substrate into the dielectric, resulting in the formation of undesirable low-permittivity silicates and the consequent degradation of the EOT value. In this section, a successfully fabricated gate-first and SCE-tolerant n-MOSFET with deep sub-nm EOT that uses both TiN/LaAlO_3_ gate stack and Schottky source/drain technologies will be demonstrated [37].

The n-MOSFETs used in this study were fabricated as follows. LaAlO_3_ gate dielectric films were deposited on LOCOS-isolated Si(100) wafers at 600 °C by PLD after dilute HF treatment. TiN film with a thickness of 60 nm was deposited as a gate electrode material on the LaAlO_3_ film by the sputtering method. The TiN gate pattern was formed by reactive ion etching (RIE) with the conditions specified in reference [38]. After forming the gate pattern, an SiO_2_ side wall with a thickness of 9.6 nm was formed on the wafers at 380 °C by atmospheric pressure plasma chemical vapor deposition. Then, the SiO_2_ layers on the gate and source/drain area were etched by RIE using CHF_3_ plasma. After wet etching pretreatment using dilute HCl, NiSi-salicide was formed by depositing 20 nm of Ni by the sputtering method followed by rapid thermal annealing in an N_2_ ambient at 450 °C for 1 min. Unreacted Ni was removed by wet etching using a 1:2 H_2_O_2_:H_2_SO_4_ solution. The wafers were then implanted with sulfur ions (implantation energy: 23 keV, fluence: 2 × 10^15^/cm^2^). The implantation energy of 23 keV was selected to adjust the projection range (Rp) so that the center of the NiSi layer (in the depth direction) was set as the target depth for implantation. After implantation, drive-in annealing for the implanted sulfur was performed in an N_2_ ambient for 1 min at 450 °C [39]. The substrate impurity concentration was estimated from C-V measurements to be 1.6 × 10^14^/cm^3^. In order to clarify the effect of the Schottky source/drain on the SCE immunity for a conventional MOSFET, no additional implantation, such as channel implantation or halo implantation, was performed.

### 6.1. Successful Fabrication of Gate-First n-MOSFET with Deep Sub-nm EOT

**Figure 40:**
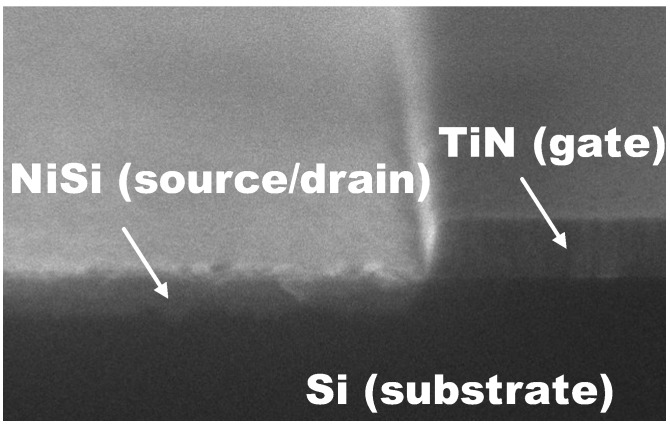
Perspective-view SEM image of an n-MOSFET incorporating both metal gate/high-*k* gate stack and sulfur-implanted Schottky source/drain technologies.

**Figure 41:**
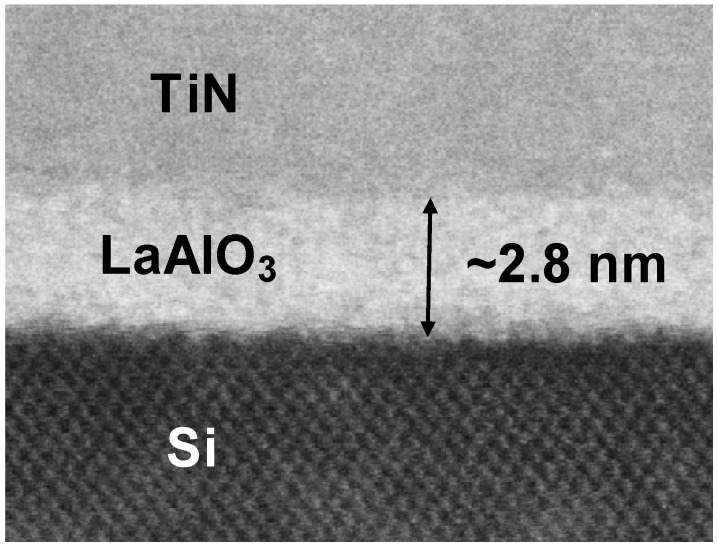
Cross-sectional STEM image of a metal gate/high-*k* (TiN/LaAlO_3_) gate stack.

Figure 40 shows a scanning electron microscopy (SEM) image of the fabricated n-MOSFET (perspective view). It was confirmed that the gate pattern was successfully fabricated with a taper angle of almost 90° and that no severe agglomeration of NiSi could be observed. AES depth-profile analysis revealed that nickel monosilicide was formed in the source/drain region. Figure 41 shows a cross-sectional scanning transmission electron microscopy (STEM) image of the TiN/LaAlO_3_/Si gate stack. The physical film thickness of LaAlO_3_ was estimated to be about 2.8 nm. As expected, thanks to the low-temperature fabrication process, no interfacial layer of SiO_2_ or silicate was observed in the gate stack.

**Figure 42:**
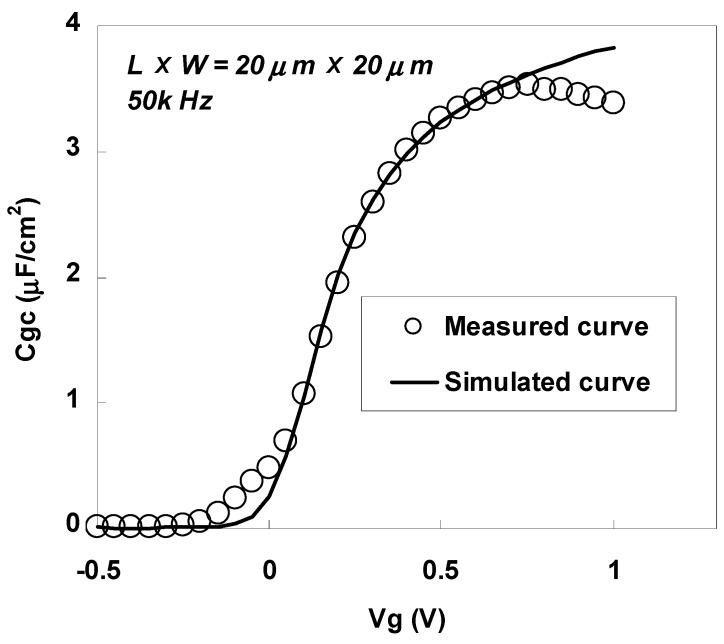
Cgc-Vg characteristics for an n-MOSFET annealed at 450 °C after sulfur implantation.

Figure 42 shows the gate-to-channel capacitance (Cgc)-Vg characteristics for an n-MOSFET with LXW = 20 μm × 20 μm, which was annealed at 450 °C after sulfur implantation. The measurement was performed at a frequency of 50 kHz. A fitted simulation curve is also shown in the figure [8]. The measured capacitance was observed to be lossy in the high electric field region due to the high gate leakage current. However, the inversion thickness and EOT estimated by comparison with the simulated curve were as small as 0.90 nm and 0.58 nm, respectively. The obtained EOT was smaller than that reported in a previous study on TaN/LaTiO n-MOSFETs using the gate-first process [40]. These small values of the inversion thickness and EOT are inherent to the LaAlO_3_ high-*k* dielectric, but cannot be achieved when conventional source/drain technology is used for the gate-first process, because the Si atoms from the substrate easily diffuse into the LaAlO_3_ layer during high-temperature annealing for the formation of pn junctions, resulting in undesirable lowering of the permittivity.

### 6.2. Comparison of MOSFET Characteristics between Sulfur-Implanted Schottky Source/Drain MOSFETs and Conventional MOSFETs

**Figure 43:**
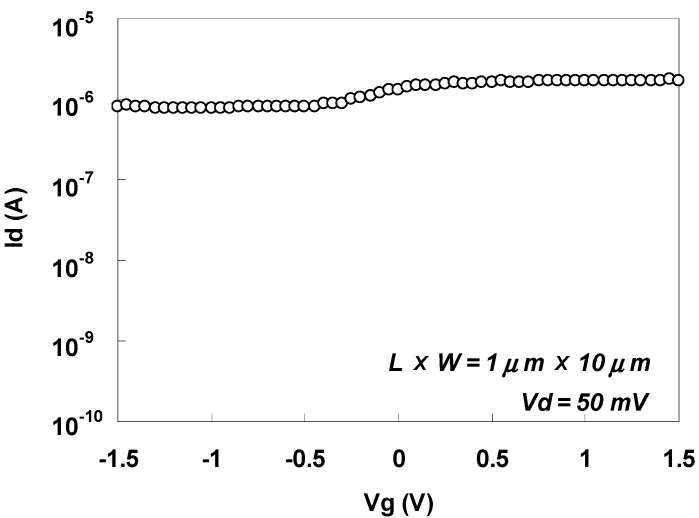
Id-Vg characteristics for an n-MOSFET immediately after implantation of sulfur.

Figure 43 shows the Id-Vg characteristics immediately after implantation of sulfur for an n-MOSFET with LXW = 1 μm × 10 μm. As can be seen in the figure, off-leakage suppression was small, resulting in a small Ion/Ioff ratio. The main component of the large off-leakage current was the reverse current of the Schottky diode because the NiSi/p-Si Schottky barrier height for holes was small. This behavior is similar to that before sulfur implantation (data not shown).

**Figure 44:**
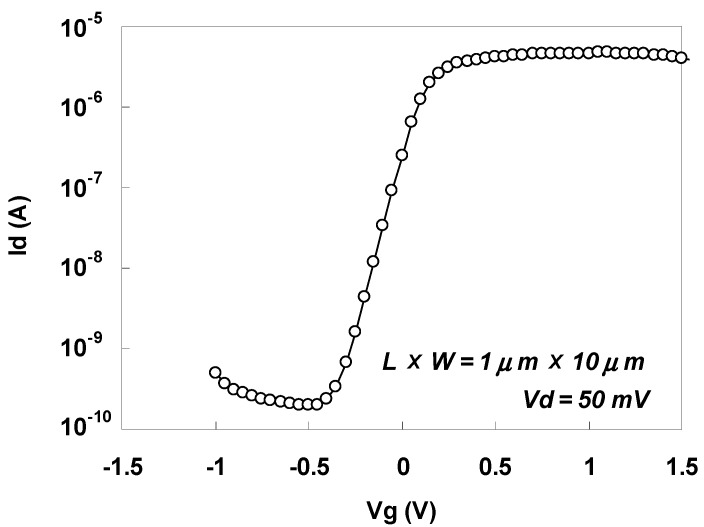
Id-Vg characteristics for an n-MOSFET annealed for 1 min at 450 °C after sulfur implantation.

The effect of sulfur implantation can be seen only when it diffuses into the NiSi/Si interface as a result of drive-in annealing followed by modulation of the NiSi/Si Schottky barrier height [39,41]. Figure 44 shows the Id-Vg characteristics for an n-MOSFET annealed for 1 min at 450 °C after sulfur implantation. As can be seen by comparing Figure 44 with Figure 43, both the drive-current and the suppression of off-leakage were dramatically improved by drive-in annealing, and well-behaved Id-Vg characteristics were obtained.

**Figure 45:**
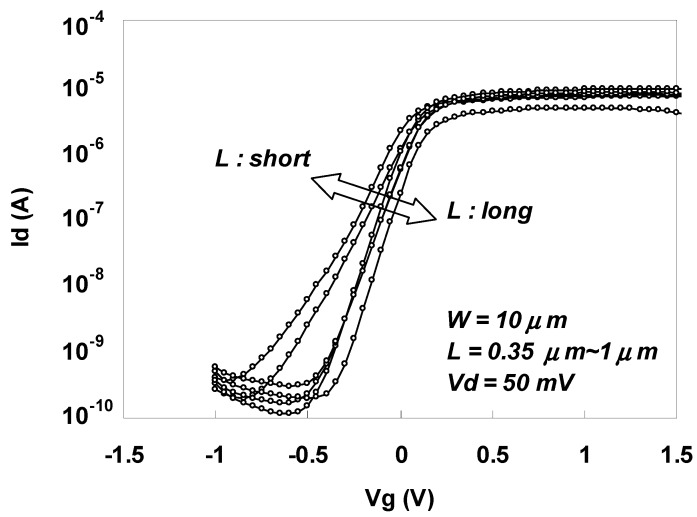
Id-Vd characteristics for an n-MOSFET annealed 1 minat 450 °C after sulfur implantation.

Figure 45 shows the Id-Vg characteristics for gate lengths ranging from 0.35 μm to 1.0 μm for n-MOSFETs annealed for 1 min at 450 °C after sulfur implantation. The characteristics were well behaved as a whole, and a current on/off ratio of more than 4 orders of magnitude was obtained.

The main performance advantage of Schottky source/drain contacts is the immunity against SCE due to a shallower junction. Therefore, the minimum channel length (L_min_) for which long channel subthreshold behavior can be observed was investigated using the gate length (L) dependence of Vth (Vth roll-off characteristics) of the n-MOSFET with Schottky source/drain used in this study. The estimated L_min_ was compared with that of a conventional MOSFET obtained from the following well-known empirical relationship [42]:

L_min_ = 0.4 × [r_j_ × d × (Ws + Wd)^2^]^1/3^ (3)

where r_j_ is the junction depth, d is the equivalent oxide thickness, and (Ws + Wd) is the sum of source and drain depletion widths.

For the n-MOSFET with Schottky source/drain used in this study, r_j_ was around 48 nm (average value obtained from the cross-sectional SEM image), d was 0.58 nm as mentioned earlier, and (Ws + Wd) was 5.23 μm (calculated from the substrate impurity concentration and applied drain voltage).

**Figure 46:**
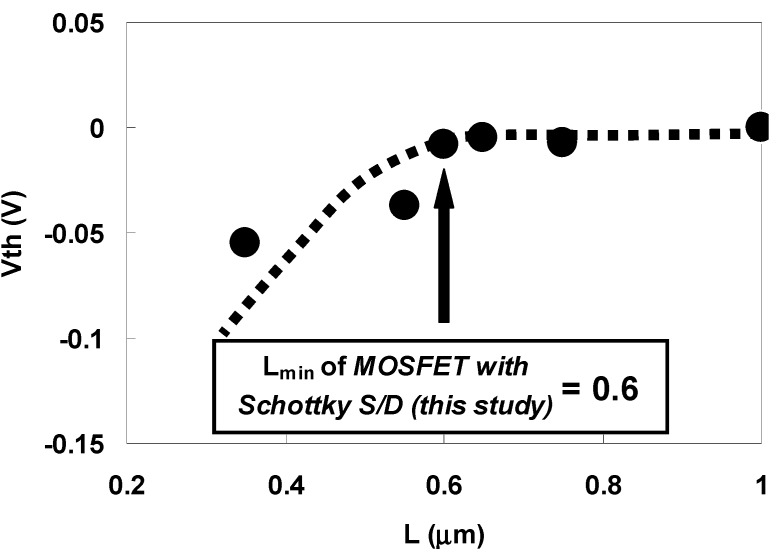
Id-Vg characteristics for gate lengths ranging from 0.35 μm to 1.0 μm for n-MOSFETs annealed for 1 min at 450 °C after sulfur implantation.

Figure 46 shows the gate length (L) dependence of Vth (Vth roll-off characteristics) for the sulfur-implanted n-MOSFET with Schottky source/drain. Note that Vth is normalized against its value at a gate length of 1 μm for a clearer comparison. As can be seen in the figure, a significant negative Vth shift due to the dependence of SCE on the gate length was clearly observed when L was less than 0.6 μm, indicating that L_min_ for the Schottky source/drain n-MOSFET used in this study was around 0.6 μm. On the other hand, based on Equation (3), a shallow rj of 29 nm is needed in a conventional MOSFET to obtain the same L_min_ as that for a Schottky source/drain MOSFET with an rj of 48 nm. This result clearly demonstrates the advantage of n-MOSFETs that use Schottky source/drain contacts over n-MOSFETS that use conventional source/drain contacts.

## 7. Conclusions

The electrical and physical characteristics of LaAlO_3_ gate dielectrics for advanced CMOS devices have been comprehensively studied. An ultrathin EOT without any interfacial layer was fabricated and its thermal stability at the interface with Si was demonstrated. The direct bonding with Si was revealed to cause greater tensile strain at the Si interface compared to when an SiO_2_ interfacial layer was present. The advantages of LaAlO_3_ gate dielectrics over Hf-based high-*k* dielectrics include thermal stability at the Si interface and stability of the effective work function. The effective work function of Lanthanum Aluminate film can be tuned over a wide range by controlling the La/(La + Al) atomic ratio when an SiO_2_ layer is inserted. Furthermore, the compatibility with the gate-first process has been demonstrated using a low-temperature process for fabrication of the sulfur-implanted Schottky source/drain. The findings of this study show the great potential of LaAlO_3_ gate dielectrics as candidates to succeed Hf-based high-*k* materials in advanced CMOS devices.

## References

[1] Mistry K., Allen C., Auth C., Beattie B., Bergstrom D., Bost M., Brazier M., Buehler M., Cappellani A., Chau R. A 45 nm logic technology with High-*k* + metal gate transistors, strained silicon, 9 Cu interconnect layers, 193 nm dry patterning, 100% Pb-free packaging. Proceedings of IEEE International Electron Devices Meeting.

[2] (2010). International Technology Roadmap for Semiconductors.

[3] Wu Y.H., Yang M.Y., Chin A., Chen W.J., Kwei C.M. (2000). Electrical characteristics of high quality La_2_O_3_ gate dielectric with equivalent oxide thickness of 5 Å. IEEE Trans. Electron. Devices.

[4] Lu X.-B., Liu Z.-G., Wang Y.-P., Yang Y., Wang X.-P., Zhou H.-W., Nguyen B.-Y. (2003). Structure and dielectric properties of amorphous LaAlO_3_ and LaAlOxNy films as alternative gate dielectric materials. J. Appl. Phys..

[5] Suzuki M., Yamaguchi T., Fukushima N., Koyama M. (2008). LaAlO_3_ gate dielectric with ultrathin equivalent oxide thickness and ultralow leakage current directly deposited on Si substrate. J. Appl. Phys..

[6] Shirley D.A. (1972). High-resolution x-ray photoemission spectrum of the valence bands of gold. Phys. Rev. B.

[7] Yang K.J., Hu C. (1999). MOS capacitance measurements for high-leakage thin dielectrics. IEEE Trans. Electron. Devices.

[8] Yasuda N., Yamaguchi T., Nishikawa Y., Satake H., Fukushima N. Composition of ideal C-V curves for ultrathin gate dielectrics based on experimental determination of substrate surface capacitance and potential. Proceedings of the Extended Abstracts of International Workshop on Gate Insulator (IWGI 2001).

[9] Gendt S.D., Chen J., Carter R., Cartier E., Caymax M., Claes M., Conard T., Delabie A., Deweerd W., Kaushik V. Implementation of high-*k* gate dielectrics—A status update. Proceedings of the Extended Abstracts of International Workshop on Gate Insulator (IWGI 2003).

[10] Koike M., Ino T., Kamimuta Y., Koyama M., Kamata Y., Suzuki M., Mitani Y., Nishiyama A., Tsunashima Y. Effect of Hf–N bond on properties of thermally stable amorphous HfSiON and applicability of this material to sub-50 nm technology node LSIs. Proceedings of the IEEE International Electron Devices Meeting (IEDM’ 03).

[11] Gardner M.I., Gopalan S., Gutt J., Peterson J., Li H.-J., Huff H.R. EOT scaling and device issues for high-*k* gate dielectrics. Proceedings of the Extended Abstracts of International Workshop on Gate Insulator (IWGI 2003).

[12] Lee S.J., Luan H.F., Bai W.P., Lee C.H., Jeon T.S., Senzaki Y., Roberts D., Kwong D.L. High-quality ultrathin CVD HfO gate stack with Poly-Si gate electrode. Proceedings of the International Electron Devices Meeting.

[13] Choi C., Kang C.Y., Rhee S.J., Abkar M.S., Krishna S.A., Zhang M., Kim H., Lee T., Zhu F., Ok I. Fabrication of TaN-gated ultra-thin MOSFETs (EOT < 1.0 nm) with HfO_2_ using a novel oxygen scavenging process for Sub 65 nm application. Proceedings of the 2005 Symposium on VLSI Technology.

[14] Migita S., Morita Y., Mizubayashi W., Ota H. Preparation of epitaxial HfO_2_ Film (EOT = 0.5 nm) on Si substrate using atomic-layer deposition of amorphous film and rapid thermal crystallization (RTC) in an abrupt temperature gradient. Proceedings of the International Electron Devices Meeting.

[15] Takahashi M., Ogawa A., Hirano A., Kamimuta Y., Watanabe Y., Iwamoto K., Migita S., Yasuda N., Ota H., Nabatame T., Toriumi A. Gate-first processed FUSI/HfO_2_/HfSiO_X_/Si MOSFETs with EOT = 0.5 nm. Proceedings of the International Electron Devices Meeting.

[16] Huang J., Heh D., Sivasubramani P., Kirsch P.D., Bersuker G., Gilmer D.C., Quevedo-Lopez M.A., Hussain M.M., Majhi P., Lysaght P. Gate first high-*k*/metal gate stacks with zero SiOx interface achieving EOT = 0.59 nm for 16 nm application. Proceedings of the Symposium on VLSI Technology.

[17] Ragnarsson L.-Å., Chiarella T., Togo M., Schram T., Absil P., Hoffmann T. (2011). Ultrathin EOT high-j/metal gate devices for future technologies: Challenges, achievements and perspectives. Microelectron. Eng..

[18] Edge L.F., Schlom D.G., Chambers S.A., Cicerrella E., Freeouf J.L., Holländer B., Schubert J. (2004). Measurement of the band offsets between amorphous LaAlO_3_ and silicon. Appl. Phys. Lett..

[19] Li A.-D., Shao Q.-Y., Ling H.-Q., Cheng J.-B., Wu D., Liu Z.-G., Ming N.-B., Wang C., Zhou H.-W., Nguyen B.-Y. (2000). Characteristics of LaAlO_3_ gate dielectrics on Si grown by metalorganic chemical vapor deposition. Appl. Phys. Lett..

[20] Yu H.Y., Li M.F., Cho B.J., Yeo C.C., Joo M.S., Kwong D.-L., Pan J.S., Ang C.H., Zheng J.Z., Ramanathan S. (2002). Energy gap and band alignment for (HfO_2_)_x_(Al_2_O_3_)_1−x_ on (100) Si. Appl. Phys. Lett..

[21] Suzuki M., Koyama M. (2009). Strain enhancement in Si induced by direct bonding of a LaAlO_3_ film to a Si substrate. Nucl. Instr. Meth. B.

[22] Kimura K., Ohshima K., Mannami M. (1994). Monolayer analysis in Rutherford backscattering spectroscopy. Appl. Phys. Lett..

[23] Nakajima K., Joumori S., Suzuki M., Kimura K., Osipowicz T., Tok K.L., Zheng J.Z., See A., Zhang B.C. (2003). Strain profiling of HfO_2_/Si(001) interface with high-resolution Rutherford backscattering spectroscopy. Appl. Phys. Lett..

[24] Chan T.K., Darmawan P., Ho C.S., Malar P., Lee P.S., Osipowicz T. (2008). Interface strain study of thin Lu_2_O_3_/Si. Nucl. Instr. Meth. B.

[25] Suzuki M., Takashima A., Koyama M., Iijima R., Ino T., Takenaka M. (2004). Characterization of Si(100)/HfSiON interface. Nucl. Instr. Meth. B.

[26] Cartier E., McFeely F.R., Narayanan V., Jamison P., Linder B.P., Copel M., Paruchuri V.K., Basker V.S., Haight R., Lim D. Role of oxygen vacancies in VFB/Vt stability of pFET metals on HfO_2_. Proceedings of the Symposium on VLSI Technology.

[27] Schaeffer J.K., Fonseca L.R.C., Samavedam S.B., Liang Y., Tobin P.J., White B.E. (2004). Contributions to the effective work function of platinum on hafnium dioxide. Appl. Phys. Lett..

[28] Tsuchiya Y., Koyama M. (2006). Work function instability at pMOS metal/HfSiON interfaces. Solid State Devices Mater..

[29] Suzuki M., Kinoshita A., Schimizu T., Koyama M. (2009). Investigation of stability of the effective work function on LaAlO_3_ and La_2_Hf_2_O_7_. J. Appl. Phys..

[30] Dimoulas A., Vellianitis G., Mavrou G., Apostolopoulos G., Travlos A., Wiemer C., Fanciulli M., Rittersma Z.M. (2004). La_2_Hf_2_O_7_ high-*k* gate dielectric grown directly on Si(001) by molecular-beam epitaxy. Appl. Phys. Lett..

[31] Yamamoto Y., Kita K., Kyuno K., Toriumi A. Study of La concentration dependent VFB shift in metal/HfLaOx/Si capacitors. Proceedings of the International Conference on Solid State Devices Materials.

[32] Kang C.Y., Kirsch P., Heh D., Young C., Sivasubramani P., Bersuker G., Song S.C., Choi R., Lee B.H., Lichtenwalner J. nMOSFET reliability improvement attributed to the interfacial dipole formed by La incorporation in HfO_2_. Proceedings of the International Conference on Solid State Devices and Materials (SSDM).

[33] Kamiyama S., Miura T., Kurosawa E., Kitajima M., Ootuka M., Aoyama T., Nara Y. Band edge gate first HfSiON/metal gate n-MOSFETs using ALD-La_2_O_3_ cap layers scalable to EOT = 0.68 nm for hp 32 nm bulk devices with high performance and reliability. Proceedings of the IEEE International Electron Devices Meeting (IEDM 2007).

[34] Kamimuta Y., Iwamoto K., Nunoshige Y., Hirano A., Mizubayashi W., Watanabe Y., Migita S., Ogawa A., Ota H., Nabatame T. Comprehensive study of VFB shift in high-*k* CMOS—Dipole formation, Fermi-level pinning and oxygen vacancy effect. Proceedings of the IEEE International Electron Devices Meeting (IEDM 2007).

[35] Suzuki M., Koyama M., Kinoshita A. (2009). Effect of composition in ternary La-Al-O films on flat-band voltage for application to dual high-*k* gate dielectric technology. Jpn. J. Appl. Phys..

[36] Jin H., Cho Y.J., Oh S.K., Kang H.J., Park J.C., Heo S., Lee J.C. (2008). Band gap engineering for La aluminate dielectrics on Si (100). Appl. Phys. Lett..

[37] Suzuki M., Nishi Y., Kinoshita A. (2011). Gate-first metal-gate/high-*k* n-MOSFETs with deep sub-nm equivalent oxide thickness (0.58 nm) fabricated with sulfur-implanted schottky source/drain using a low-temperature process. IEEE Trans. Electron Devices.

[38] Tonotani J., Iwamoto T., Sato F., Hattori K., Ohmi S., Iwai H. (2003). Dry etching characteristics of TiN film using Ar/CHF_3_, Ar/Cl_2_, and Ar/BCl_3_ gas chemistries in an inductively coupled plasma. J. Vac. Sci. Technol..

[39] Nishi Y., Kinoshita A. NiSi metal S/D transistors with ultimately low schottky barrier by sulfur implantation after Silicidation process. Proceedings of the International Conference on Solid State Devices Materials.

[40] Lin S.H., Cheng C.H., Chen W.B., Yeh F.S., Chin A. (2009). Low-threshold-voltage TaN/LaTiO n-MOSFETs with small EOT. IEEE Electron Device Lett..

[41] Zhao Q.T., Breuer U., Rije E., Lenk St., Mantl S. (2005). Tuning of NiSi/Si Schottky barrier heights by sulfur segregation during Ni silicidation. Appl. Phys. Lett..

[42] Brews J.R., Fichtner W., Nicollian E.H., Sze S.M. (1980). Generalized guide for MOSFET miniaturization. IEEE Electron Device Lett..

